# Reactivity of Rh(I) Complexes Toward Fluorinated Ethers: Activation of Inhalation Anesthetics

**DOI:** 10.1002/open.70328

**Published:** 2026-11-01

**Authors:** Soodeh Mollasalehi, Jakob Bölle, Thomas Braun

**Affiliations:** ^1^ Department of Chemistry Humboldt‐Universität zu Berlin Berlin Germany

**Keywords:** C─F bond activation, fluoroethers, hydrodehalogenation, inhalation anesthetics, rhodium

## Abstract

The reactivity of [Rh(H)(PEt_3_)_3_] (**1**) toward α‐fluorinated ethers that are commonly used as inhalational anesthetics was studied. Unprecedented reactivity pathways include hydrodehalogenation of *sp* ^3^ C─F and C─Cl bonds under mild conditions. The reaction of **1** with sevoflurane (CF_3_)_2_CHOCH_2_F provides an efficient route to obtain [Rh(F)(PEt_3_)_3_] (**2**) by C(*sp* ^3^)─F bond activation. Furthermore, the phosphine ligand takes part in this reaction, and an F/Et exchange at [Rh(H)(F)_2_(PEt_3_)_3_] results in the formation of the fluorophosphine complex *trans*‐[Rh(F)(Et_2_PF)(PEt_3_)_2_] (**5**) upon the release of ethane. On the other hand, an oxidative addition of enflurane CClFHCF_2_OCF_2_H at **1** yielded [Rh(H)(Cl)(CFHCF_2_OCF_2_H)(PEt_3_)_3_] (**8**), which is followed by a reductive elimination step to yield [Rh(Cl)(PEt_3_)_3_] (**7**) and the hydrofluoroether CFH_2_CF_2_OCF_2_H. When an isomer of enflurane, isoflurane CF_3_CClHOCF_2_H, was treated with **1**, complex **7** and CF_3_CH_2_OCF_2_H are generated by C(*sp* ^3^)─Cl bond activation. Additionally, an in situ nucleophilic attack of triethylphosphine at the hydrofluoroether CF_3_CH_2_OCF_2_H gave a unique fluorinated phosphonium salt [CF_3_CH_2_PEt_3_]_2_[SiF_6_] (**9**). Catalytic hydrodechlorination of isoflurane at a mild condition was achieved in the presence of silane HSiPh_3_ using **1** as a catalyst.

## Graphical abstract

Reactions of the inhaled anesthetics and α‐fluorinated ethers sevoflurane, enflurane, and isoflurane with [Rh(H)(PEt_3_)_3_] led to versatile organometallic transformations including C(sp^3^)─F and C(sp^3^)─Cl bond cleavage and metal–halogen bond formation, a catalytic hydrodehalogenation reaction, as well as the generation of a unique fluorinated phosphonium salt.

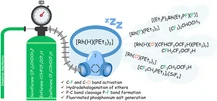

## 1 Introduction

Fluorine has been widely exploited in a broad range of fields such as pharmaceutics, agrochemicals, materials, refrigeration, and drug discovery [1, 2]. Modification of the acidity, the lipophilicity, the conformations, and the metabolism of drugs was achieved by introducing fluorine substituents at *sp* ^3^ or *sp* ^2^ carbons [3, 4]. In the mid‐1990s, α‐fluorinated ethers were successfully developed as effective inhalational anesthetics, exhibiting significant advantageous physical properties [5, 6].

The exceptionally high strength of the C─F bond contributes to the stability of fluorinated anesthetics [7]. Additionally, the pronounced polarity of carbon−fluorine bonds may promote interactions between fluorinated agents and the proteins that mediate their anesthetic activity [8]. The introduction of fluorine and other halogens on ether molecules increases volatility and generally decreases toxicity and flammability [9, 10]. The incorporation of halogens also reduces blood–gas solubility of inhalants, thereby the recovery time is shortened [11].

Among others, the α‐fluorinated ethers enflurane (2‐chloro‐1‐(difluoromethoxy)‐1,1,2‐trifluoroethane), isoflurane (2‐chloro‐2‐(difluoromethoxy)‐1,1,1‐trifluoroethane), and sevoflurane (1,1,1,3,3,3‐hexafluoro‐2‐(fluoromethoxy)propane) have been clinically used for general anesthesia since the early 1970s [12]. Despite the highly desirable medical properties of modern halogenated anesthetics, they remain a subject of debate because of their contributions to the greenhouse effect and ozone depletion [13]. They undergo minimal metabolic transformation during clinical application and volatilize almost entirely into the atmosphere, leading to rapid global accumulation [14]. Consequently, their high global warming potentials (GWP) have raised increasing environmental concerns, prompting several health services to begin phasing down their use [15, 16]. However, there is currently no feasible alternative for replacing them in daily routine [17]. Instead, because of the relative ease of bulk production of some of these chemicals, there are potential benefits in using them as starting points for developing new fluorinated building blocks.

Studies on the reactivity of fluorinated compounds toward transition‐metal complexes, main‐group complexes, or heterogeneous catalysts have been widely carried out [18, 19, 20]. At rhodium, complexes [Rh(E)(PEt_3_)_3_] (E = H, boryl, germyl, and silyl) successfully promote C─F bond activation of hydrofluoroolefins and hydrofluoroaromatics, driven by the formation of stable E─F bonds that lead to either metal−carbon or metal−fluorine bond formation [21, 22, 23, 24, 25].

Hydrodehalogenation is an effective strategy for degrading environmentally persistent halocarbons and is therefore of strong industrial and synthetic relevance [26, 27, 28, 29]. It is also a well‐established reaction pathway at transition‐metal complexes, widely applied in both stoichiometric and catalytic activation of fluorinated substrates [30, 31, 32]. Catalytic dehalogenation research has predominantly addressed aryl and vinyl halides, whereas alkyl halides, which possess stronger bonds, remain comparatively underexplored [33, 34, 35]. Notably, catalysts that perform efficiently with aromatic substrates often show little or no activity toward aliphatic counterparts [29]. Along these lines, studies on the activation and functionalization of fluorine‐containing ethers are very scarce.

Noticeably, we established a metal‐ and phosphine‐mediated decarbonylation of perfluoro(methyl vinyl ether) at rhodium (I) phosphine complexes. [36, 37] In another recent work, the degradation of a polyfluoroalkyl ether using aluminum‐based Lewis acids was reported [38]. The group of Friesen described the use of methyl hydrofluoroethers as electrophilic methylating agents of tertiary amines and trimethylphosphine to afford quaternary ammonium or phosphonium perfluoroalkoxides, respectively [39]. Huang and coworkers described the dehydrofluorination of sevoflurane using organolithium bases [40, 41]. Recently, the group of Crimmin utilized fluoroethers as the fluorine source in a transfer fluorination procedure using the *p*‐tolylsulfonyl chloride as a fluorine acceptor [15].

Herein, we present the reactivity of α‐fluorinated ethers toward [Rh(H)(PEt_3_)_3_] (**1**). The reaction routes include C(*sp* ^3^)─‍‍F and C(*sp* ^3^)─Cl bond cleavage accompanied by metal–halogen bond formation. The formation of new hydrofluoroether building blocks proceeds through hydrodehalogenation pathways and rhodium (III) intermediate species. Selective catalytic hydrodechlorination of a halogenated ether with HSiPh_3_ is achieved using complex **1** as the catalyst.

## 2 Results and Discussion

The reaction of an excess amount of sevoflurane (CF_3_)_2_CHOCH_2_F with [Rh(H)(PEt_3_)_3_] (**1**) led after 36 h at 55 °C to the formation of the fluorido complex [Rh(F)(PEt_3_)_3_] (**2**) and (CF_3_)_2_CHOCH_3_ (Scheme 1). In addition to **2**, *trans*‐[Rh(F)(CO)(PEt_3_)_2_] (**3**) and *cis*‐*fac*‐[Rh(H)_2_(F)(PEt_3_)_3_] (**4**) (in a ratio of 1:0.3:0.2) were identified by NMR spectroscopy. When the reaction mixture was heated further, a smooth conversion of **2** and **4** into the fluorophosphine complex *trans*‐[Rh(F)(Et_2_PF)(PEt_3_)_2_] (**5**) and ethane was observed. Complex **4** has been reported to be synthesized by treating **1** with an HF source [42]. Additionally, a further conversion of **2** into **3** was observed over time. A full characterization of the complexes **2**, **3**, and **4** has been reported in the literature [42, 43]. Note that our group recently demonstrated the role of perfluoro(methyl vinyl ether) as a source of CO in the reaction with rhodium complexes, leading to the generation of carbonylation products such as complex **3** [37].

**SCHEME 1:**
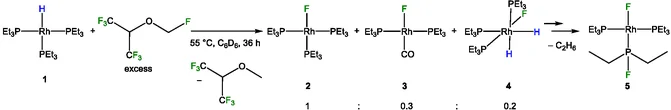
Reactivity of sevoflurane toward [Rh(H)(PEt_3_)_3_] (**1**).

Complex **5** was characterized by ^1^H, ^31^P{^1^H}, and ^19^F NMR spectroscopy. The ^31^P{^1^H} NMR spectrum exhibits two resonances of inequivalent phosphorus atoms at 220.4 and 25.8 ppm in a ratio of 1:2, which are mutually coupled by ^2^ *J*_P,P_ = 41.6 Hz. The low‐field‐shifted signal at 220.4 ppm appears as a doublet of doublet of doublet of triplets due to the coupling to the phosphorus‐bounded fluorine atom (^1^ *J*_P,F_ = 818.3 Hz), to the *trans* fluorido ligand (^2^ *J*_P,F_ = 216.2 Hz), to rhodium (^1^ *J*_P,Rh_ = 208.0 Hz), and to the other phosphines. The second resonance corresponding to the phosphine ligands in mutually *trans* position appears at 25.8 ppm as a doublet of doublet of triplets due to the coupling to rhodium (^1^ *J*_P,Rh_ = 136.1 Hz), to phosphorus, to the phosphorus‐bounded fluorine atom, and to the fluorido ligand (^2^ *J*_P,F_ ~ ^3^ *J*_P,F_ = 26.2 Hz). In the ^19^F NMR spectrum, the fluorido ligand signal resonates at −261.1 ppm as a doublet of doublet of triplet of doublets due to the coupling to the phosphine in the *trans*position, to rhodium (^1^ *J*_F,Rh_ = 42 Hz), to the *cis* phosphines, and to the other fluorine atom (^3^ *J*_F,F_ = 12 Hz). The signal at −132.3 ppm displays a doublet of triplet of doublet of doublets due to the coupling to the directly bounded phosphorus atom, to the *cis* phosphines, to rhodium (^2^ *J*_F,Rh_ = 20 Hz), and to the fluorido ligand.

Note that the formation of a phenyl analog of complex **5**, *trans*‐[Rh(F)(Ph_2_PF)(PPh_3_)_2_], has been reported in the literature, and it is generated upon heating the fluorido congener of Wilkinson’s catalyst [44, 45, 46]. Upon heating the Ir(I) methyl complex [Ir(CH_3_)(PEt_3_)_3_] in hexafluorobenzene, a C─F bond activation reaction yielded the fluorophosphine complex *trans*‐[Ir(C_6_F_5_)(Et_2_PF)(PEt_3_)_2_] together with methane and ethylene [47]. Additionally, C─F bond activation of pentafluoropyridine at Pt(0) complexes [Pt(PR_3_)_2_] (R = *i*Pr, Cy CHMe_2_) led to the formation of [Pt(4‐C_5_NF_4_)(R)(R_2_PF)(PR_3_)] [48].

A plausible mechanistic pathway for the transformation of complex **2** into **5** involves the dimerization of **2** to give [{Rh(µ‐F)(PEt_3_)_2_}_2_] (**A**) and the free triethylphosphine (Scheme 2). The oxidative addition of HF at the phosphine yields the phosphorane species PEt_3_(H)(F), which together with **A** forms the metallophosphorane rhodium(I) complex [Rh(H)(F)(Et_3_PF)(PEt_3_)_2_] (**B**). The migration of the ethyl group to the metal center leads to the formation of [Rh(H)(F)(C_2_H_5_)(Et_2_PF)(PEt_3_)_2_] (**C**), which undergoes a reductive elimination of ethane to finally yield complex **5**. It is also noteworthy that, in the presence of HF sources, complex **2** can not only form the binuclear fluorido complex **A** but also the bifluorido complex [Rh(FHF)(PEt_3_)_3_] (**D**), as described in the literature [24]. As mentioned above, the formation of complex **4** includes the oxidative addition of HF at complex **1**, whereas the oxidative addition of HF at complex **2** has not been observed previously. Nevertheless, the possible formation of the difluorido hydrido rhodium complex [Rh(H)(F)_2_(PEt_3_)_3_] upon further reaction of **2** cannot be entirely excluded. This intermediate could then undergo an F/Et rearrangement to afford complex **5**, accompanied by the reductive elimination of ethane, as postulated by Grushin and Marshall for the rhodium fluorido complex *trans*‐[Rh(F)(Ph_2_PF)(PPh_3_)_2_] [44].

**SCHEME 2:**
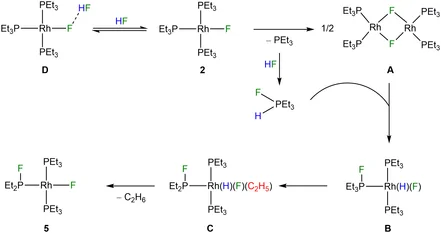
Mechanistic proposal for the conversion of complex **2** to complex **5**.

Treatment of [Rh(H)(PEt_3_)_3_] (**1**) with an excess amount of enflurane CClFHCF_2_OCF_2_H at room temperature provided [Rh(Cl)(PEt_3_)_3_] (**7**) and difluoromethyl 1,1,2‐trifluoroethyl ether, CFH_2_CF_2_OCF_2_H, via hydrodechlorination (Scheme 3). The intermediate [Rh(H)(Cl)(CFHCF_2_OCF_2_H)(PEt_3_)_3_] (**8**) was identified by NMR spectroscopy after 5 min as product of an oxidative addition of C(*sp* ^3^)─Cl bond of the ether at complex **1**. Monitoring the reaction progress by NMR spectroscopy revealed the transformation of **8** into the rhodium chlorido complex **7** and CFH_2_CF_2_OCF_2_H. Note that the catalytic dehydrochlorination of enflurane using H_2_/Pd at 200 °C was mentioned in the literature [49].

**SCHEME 3:**
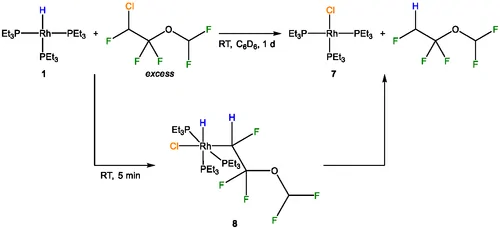
Reactivity of enflurane toward [Rh(H)(PEt_3_)_3_] (**1**).

Complex **8** is remarkably stable toward β‐fluoride elimination, possibly because this pathway requires a free coordination site that is not provided by phosphine dissociation under the reaction conditions. β‐Fluoride elimination at rhodium has, however, been described in the literature [24, 25, 50, 51, 52].

When 2.9 equivalents of enflurane were used, the conversion of **1** into **7** was completed within 24 h. Kinetic analysis was performed on the conversion of **8** into **7** and ether under conditions where **1** was fully consumed. The decay of **8** exhibits first‐order behavior with a rate constant independent of the initial concentration of enflurane (see Figure S1 for the plot of [8] vs. time).

Complex **7** and CFH_2_CF_2_OCF_2_H were identified by NMR spectroscopy, and their data have been reported previously in the literature [49, 53]. The ^31^P{^1^H} NMR spectrum of complex **8** displays two signals at 16.3 and −8.3 ppm in a 2:1 ratio with phosphorus–rhodium coupling constants (^1^ *J*_P,Rh_ = 103.0 and 85.7 Hz, respectively) and phosphorus–phosphorus coupling constants (^2^ *J*_P,P_ = 22.8 Hz) that are compatible with assignment as a Rh^lll^ complex [54, 55]. In the ^19^F NMR spectrum (Figure 1), two signals resonating as broad doublet of triplets at −71.9, −74.0 ppm can be assigned to the CF_2_ group at the α‐position to the oxygen atom with a *geminal* fluorine–fluorine coupling constant of ^2^ *J*_F,F_ = 145 Hz and coupling to the CFH group (^3^ *J*_F,F_ = 17 Hz and ^3^ *J*_F,F_ = 15 Hz, respectively). The signal at −74.0 collapses into a doublet of doublet of doublet of doublets in ^19^F{^1^H} NMR spectrum, displaying an additional coupling to the terminal CF_2_H moiety (^4^ *J*_F,F_ = 5 Hz, ^4^ *J*_F,F_ = 3 Hz), while the other signal at −71.9 collapses into doublet of doublet of pseudo triplets (^4^ *J*_F,F_ = 5 Hz). The two resonances of the terminal CF_2_H moiety appear at −83.2 and −84.3 ppm with a *geminal* fluorine–fluorine coupling constant of ^2^ *J*_F,F_ = 175 Hz, as well as *geminal* fluorine–proton coupling constant of ^2^ *J*_F,H_ = 71 Hz for both signals. The signal at −84.3 appears as a doublet of doublet of doublet of doublets due to the coupling to the above mentioned *geminal* fluorine and proton atoms, as well as the CF_2_ moiety (^4^ *J*_F,F_ = 5 Hz, ^4^ *J*_F,F_ = 3 Hz), while the signal at −83.2 exhibits a doublet of doublet of pseudo triplets (^4^ *J*_F,F_ = 5 Hz). Finally, the signal corresponding to the CFH group is shifted to higher field and appears at −171.7 ppm as a multiplet. In the ^1^H NMR spectrum, the most characteristic signal belongs to the hydrido ligand at −10.5 ppm, which appears as a doublet of quartets with a *trans* hydride−phosphine coupling constant of 183.4 Hz as well as coupling to the rhodium and the *cis* phosphines (^1^ *J*_H,Rh _~ ^2^ *J*_H,P_ = 14.4 Hz), which collapses into a doublet in the ^1^H{^31^P} NMR spectrum. The signal of the CFH group is observed in ^1^H NMR spectrum (600 MHz) at 5.9 ppm as a doublet of multiplets with a geminal fluorine–proton coupling constant of ^2^ *J*_H,F_ = 46.5 Hz. A signal corresponding to the terminal CF_2_H group resonates at 6.4 ppm as a triplet (^2^ *J*_H,F_ = 70.9 Hz), which collapses to a singlet in ^1^H{^19^F} NMR spectrum. Signal assignments were confirmed by ^1^H–^19^F COSY, ^19^F–^19^F COSY, and ^1^H‐^31^P HMBC NMR spectra (Figure 1; also see the Supporting Information). Note that the formation of a *cis*‐hydridoalkylrhodium(III) complex, *cis*‐[Rh(H)(Cl)(CH_2_COCH_3_)(PMe_3_)_3_], *via* a catalytic epoxide isomerization reaction with [Rh(Cl)(PMe_3_)_3_] is reported in the literature [56].

**FIGURE 1:**
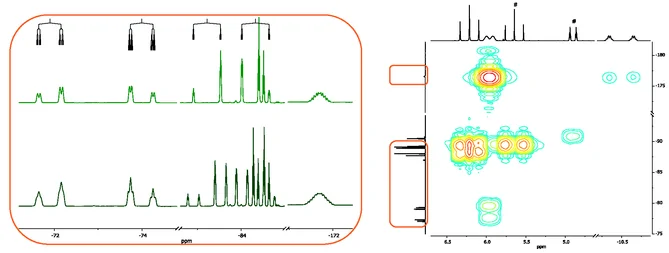
Selected sections of the ^1^H–^19^F COSY NMR spectra of **8** (right). The signals in ^19^F and ^19^F{^1^H} spectra are highlighted (left). # = excess enflurane.

Interestingly, when an isomer of enflurane, isoflurane CF_3_CClHOCF_2_H, was treated with **1**, the generation of the chlorido complex **7** and the corresponding hydrodechlorination product, 1,1,1‐trifluoro‐2‐difluoromethoxypropane, CF_3_CH_2_OCF_2_H [ 57], was observed already after 1 h at room temperature (Scheme 4). Additionally, the formation of a unique trifluoroethyl phosphonium salt [CF_3_CH_2_PEt_3_]_2_[SiF_6_] (**9**) and complex *trans*‐[Rh(Cl)(CO)(PEt_3_)_2_] (**10**) was characterized as minor side products (hydrofluoroether/**9** ratio of 10:1 and **7**/**10** ratio of 4:1 after 1 h). This observation suggests that a substitution of the phosphine with CO in complex **7** takes place, while the liberated phosphine reacts further in the reaction.

**SCHEME 4:**
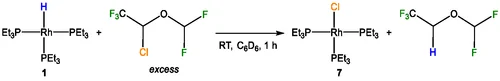
Reactivity of isoflurane toward [Rh(H)(PEt_3_)_3_] (**1**).

Mechanistically, it is feasible that the phosphine in the *trans* position to the chlorido ligand in **7** dissociates from the complex and performs a nucleophilic attack at the CH carbon at the α‐‍posi‍tion to the etheric oxygen in CF_3_CH_2_OCF_2_H to yield a trifluoroethyl phosphonium cation and difluoroalkoxide (Scheme 5). Given the instability of α‐fluoro alkoxides, it can be expected that the alkoxide spontaneously decomposes into formyl fluoride by release of a fluoride anion [58]. Transformation of formyl fluoride to HF and CO and a subsequent HF glass etching lead to the formation of hexafluorosilicate [59], which is a suitable counter anion to form the phosphonium salt **9**. Furthermore, the substitution of phosphine ligand in **7** by the produced CO yields the complex **10**.

**SCHEME 5:**
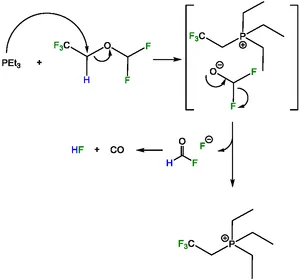
Possible mechanism for the formation of the cation in **9**, CO, HF, and fluoride.

Note that the synthesis of [CF_3_CH_2_PPh_3_][OTf] and the use of its corresponding ylide for Wittig olefination of aromatic aldehydes to give trifluoropropenyl compounds have been reported in the literature [60]. Furthermore, Hoge and coworkers developed the synthesis of halogenophosphonium salts [61, 62]. Dissociation of the phosphine ligand from complex **1** and related derivatives, followed by decarbonylation reactions in which fluoroethers act as CO sources, has previously been reported [37, 52]. Moreover, Braun and coworkers recently reported that the degradation of a polyfluoroalkyl ether can lead to the formation of CO and HF when treated with aluminum‐based Lewis acids [38].

In the ^31^P{^1^H} NMR spectrum of [CF_3_CH_2_PEt_3_]_2_[SiF_6_] (**9**), a signal at 37.1 ppm appears as a quartet due to the coupling of the phosphorus atom to the fluorine atoms of the CF_3_ group. The corresponding fluorine signal to the CF_3_ group was observed at −55.5 ppm in the ^19^F NMR spectrum as a triplet of doublets due to the coupling to the CH_2_ group and to the phosphorus atom (^3^ *J*_F,P_ = 11, ^3^ *J*_F,H_ = 6 Hz). Indeed, this signal collapses into a doublet in the ^19^F{^1^H} NMR spectroscopy. Additionally, a broad signal at −126.4 ppm was found, which is characteristic for the fluorosilicate SiF_6_^2−^. In ^1^H NMR spectrum, a signal for the CH_2_ group appears as a doublet of quartets at 3.9 ppm (^2^ *J*_H,P_ = 14.2 Hz, ^3^ *J*_H,F_ = 11.5 Hz), which collapses to a quartet in ^1^H{^31^P} NMR spectrum.

Furthermore, a selective catalytic hydrodechlorination reaction of isoflurane with triphenylsilane (2.5 eq.) was achieved (Scheme 6). Thus, 10 mol% of complex **1** was used as a catalyst, and after 12 days at 58 °C, 75% conversion of the isoflurane into CF_3_CH_2_OCF_2_H was observed. The blank test reaction in the absence of catalyst **1** did not yield any product after 3 days of heating at 58 °C. Note that rhodium‐catalyzed dehalogenations of chlorinated and fluorinated ethylenes using triethylsilane have been reported [63, 64]. Interestingly, no catalytic reactivity was observed with enflurane under the same conditions. We hypothesize that the absence of a C─Cl bond in the α‐position relative to the etheric oxygen may influence the hydrodechlorination reactivity of the ethers.

**SCHEME 6:**
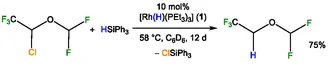
Catalytic hydrodechlorination of isoflurane with HSiPh_3_ and [Rh(H)(PEt_3_)_3_] (**1**).

## 3 Conclusion

In summary, it has been demonstrated that the rhodium(I) hydrido complex [Rh(H)(PEt_3_)_3_] (**1**) exhibits versatile reactivity pathways toward inhaled anesthetics, i.e., the α‐fluorinated ethers sevoflurane, enflurane, and isoflurane. Reactivity depends on the halogenated substitution pattern at the ether and the position of the atoms with respect to the etheric oxygen. Processes involve hydrodehalogenation of *sp* ^3^ C─F and C─Cl bonds of haloethers under mild conditions, which resulted in the formation of metal–halogen bonds and hydrofluoroether building blocks. Remarkably, a C(*sp* ^3^)─Cl bond oxidative addition of halogenated ether at **1** generated a Rh(III) intermediate bearing a unique hydrofluoroether ligand ─CFHCF_2_OCF_2_H. An unprecedented trifluoroethylation of phosphine with a hydrofluoroether yielded a unique fluorinate phosphonium salt. A selective catalytic hydrodechlorination of a halogenated ether at mild conditions is demonstrated. The study provides insights into the reactivity of inhalation anesthetics, which have shown deleterious effects on global environmental systems despite their clinical application advantageous. The reactivity pathways are characterized by unprecedented organometallic transformations. The paper additionally demonstrates that phosphines may enable useful transformations of fluorinated compounds.

## Funding

This study was supported by Deutsche Forschungsgemeinschaft (BR 2065/13−2) and Humboldt‐Universität zu Berlin.

## Conflicts of Interest

The authors declare no conflicts of interest.

## Supporting information

Supplementary Material

## Data Availability

Data are available in the Supporting Information.

## References

[1] T. Hiyama and H. Yamamoto , Organofluorine Compounds: Chemistry and Applications (Springer, 2000), 77.

[2] S. Purser , P. R. Moore , S. Swallow , and V. Gouverneur , “Fluorine in Medicinal Chemistry,” Chemical Society Reviews 37 (2008): 320, 10.1039/B610213C. 18197348

[3] O. Eisenstein , J. Milani , and R. N. Perutz , “Selectivity of C–H Activation and Competition Between C–H and C–F Bond Activation at Fluorocarbons,” Chemical Reviews 117 (2017): 8710, 10.1021/acs.chemrev.7b00163. 28653537

[4] S. Mishra , C. Jadala , S. Cardoza , G. R. Potuganti , and G. R. Velma , “Fluorine‐Containing FDA‐Approved Small‐Molecule Drugs in 2025: Significance, Synthetic Insights, and Therapeutic Applications,” RSC Advances 16 (2026): 13830, 10.1039/D6RA01711H. PMC12983373 41837169

[5] Y. Wang , X. X. Ming , and C. P. Zhang , “Fluorine‐Containing Inhalation Anesthetics: Chemistry, Properties and Pharmacology,” Current Medicinal Chemistry 27 (2020): 5599, 10.2174/0929867326666191003155703. 31580246

[6] M. Z. Hernandes , S. M. Cavalcanti , D. R. Moreira , W. F. de Azevedo Junior , and A. C. Leite , “Halogen Atoms in the Modern Medicinal Chemistry: Hints for the Drug Design,” Current Drug Targets 11 (2010): 303, 10.2174/138945010790711996. 20210755

[7] P. Jeschke , E. Baston , and F. R. Leroux , “α‐Fluorinated Ethers as “Exotic” Entity in Medicinal Chemistry,” Mini Reviews in Medicinal Chemistry 7 (2007): 1027, 10.2174/138955707782110150. 17979805

[8] J. A. Campagna , K. W. Miller , and S. A. Forman , “Mechanisms of Actions of Inhaled Anesthetics,” New England Journal of Medicine 348 (2003): 2110, 10.1056/NEJMra021261. 12761368

[9] B. K. Park , N. R. Kitteringham , and P. M. O’Neill , “Metabolism of Fluorine‐Containing Drugs,” Annual Review of Pharmacology and Toxicology 41 (2001): 443, 10.1146/pharmtox.2001.41.issue-1. 11264465

[10] J. G. Bovill , J. Schüttler , and H. Schwilden , “Inhalation Anaesthesia: From Diethyl Ether to Xenon,” in Modern Anesthetics, ed. J. Schuttler , and H. Schwilden (Springer, 2008), 121. 10.1007/978-3-540-74806-9_6 18175089

[11] D. F. Halpern , Organofluorine Chemistry: Principles and Commercial Applications (Springer, 1994), 543.

[12] E. P. Steffey , R. J. Brosnan , and K. R. Mama , “Inhalation Anesthetics,” in Veterinary Anesthesia and Analgesia: The Sixth Edition of Lumb and Jones, ed. L Lamont , K Grimm , S Robertson , L Love , and C Schroeder (Wiley Online Library, 2024), 489.

[13] S. M. Ryan and C. J. Nielsen , “Global Warming Potential of Inhaled Anesthetics: Application to Clinical Use,” Anesthesia & Analgesia 111 (2010): 92, 10.1213/ANE.0b013e3181e058d7. 20519425

[14] M. K. Vollmer , T. S. Rhee , M. Rigby , et al., “Modern Inhalation Anesthetics: Potent Greenhouse Gases in the Global Atmosphere,” Geophysical Research Letters 42 (2015): 1606, 10.1002/grl.v42.5.

[15] N. A. Jenek , S. L. Brock , J. Mao , A. A. Fogh , A. Phanopoulos , and M. R. Crimmin , “Chemical Recycling of Hydrofluorocarbons by Transfer Fluorination,” Nature Chemistry 18 (2026): 1, 10.1038/s41557-026-02096-8. PMC13149033 41826676

[16] S. M White and C. L Shelton , “Abandoning Inhalational Anaesthesia,” Anaesthesia 75 (2020): 451–454, 10.1111/anae.15148. 31531970

[17] H. Wulf , “Time to Say Goodbye to Inhalational Anaesthesia?,” Anästhesiologie & Intensivmedizin 64 (2023): 428–431.

[18] M. Ohashi and S. Ogoshi , “Catalytic Transformations of Fluorinated Olefins,” Organometallic Fluorine Chemistry 52 (2015): 197.

[19] H. Amii and K. Uneyama , “C−F Bond Activation in Organic Synthesis,” Chemical Reviews 109 (2009): 2119, 10.1021/cr800388c. 19331346

[20] V. V. Grushin , “The Organometallic Fluorine Chemistry of Palladium and Rhodium: Studies Toward Aromatic Fluorination,” Accounts of Chemical Research 43 (2010): 160, 10.1021/ar9001763. 19788304

[21] T. Braun and M. Talavera , “Rhodium(I) Complexes as Useful Tools for the Activation of Fluoroolefins,” Synlett 31 (2020): 1760, 10.1055/s-0040-1707156.

[22] A. L. Raza and T. Braun , “Consecutive C─F Bond Activation and C─F Bond Formation of Heteroaromatics at Rhodium: The Peculiar Role of FSi(OEt)_3_ ,” Chemical Science 6 (2015): 4255, 10.1039/C5SC00877H. PMC5707480 29218192

[23] M. Talavera , S. Mollasalehi , and T. Braun , “C‐H and C‐F Bond Activation of Fluorinated Propenes at Rh: Enabling Cross‐Coupling Reactions With Outer‐Sphere C‐C Coupling,” Chemical Science 15 (2024): 8472, 10.1039/D4SC00951G. PMC11151818 38846380

[24] M. Talavera and T. Braun , “Competing C‐H and C‐F Bond Activation Reactions of a Fluorinated Olefin at Rh: A Fluorido Vinylidene Complex as an Intermediate in an Unprecedented Dehydrofluorination Step,” Chemical Science 13 (2022): 1130, 10.1039/D1SC06713C. PMC8790890 35211279

[25] M. Talavera , C. N. von Hahmann , R. Muller , M. Ahrens , M. Kaupp , and T. Braun , “C‐H and C‐F Bond Activation Reactions of Fluorinated Propenes at Rhodium: Distinctive Reactivity of the Refrigerant HFO‐1234yf,” Angewandte Chemie International Edition in English 58 (2019): 10688, 10.1002/anie.v58.31. 31102565

[26] S. Liu , J. A. Otero , M. Martin‐Martinez , D. Rodriguez‐Franco , J. J. Rodriguez , and L. M. Gómez‐Sainero , “Understanding Hydrodechlorination of Chloromethanes. Past and Future of the Technology,” Catalysts 10 (2020): 1462, 10.3390/catal10121462.

[27] H. Yu , E. M. Kennedy , A. A. Adesina , and B. Z. Dlugogorski , “A Review of CFC and Halon Treatment Technologies–the Nature and Role of Catalysts,” Catalysis Surveys From Asia 10 (2006): 40, 10.1007/s10563-006-9003-z.

[28] M. A. Keane , “Supported Transition Metal Catalysts for Hydrodechlorination Reactions,” ChemCatChem 3 (2011): 800, 10.1002/cctc.v3.5.

[29] B. Sahoo , A.‐E. Surkus , M.‐M. Pohl , et al., “A Biomass‐Derived Non‐Noble Cobalt Catalyst for Selective Hydrodehalogenation of Alkyl and (Hetero)Aryl Halides,” Angewandte Chemie International Edition 56 (2017): 11242, 10.1002/anie.v56.37. 28517035

[30] C. Yao , S. Wang , J. Norton , and M. Hammond , “Catalyzing the Hydrodefluorination of CF_3_‐Substituted Alkenes by PhSiH_3_. H• Transfer From a Nickel Hydride,” Journal of the American Chemical Society 142 (2020): 4793, 10.1021/jacs.9b13757. PMC7102444 31935083

[31] H. Iwamoto , H. Imiya , M. Ohashi , and S. Ogoshi , “Cleavage of C(sp^3^)–F Bonds in Trifluoromethylarenes Using a Bis(NHC)nickel(0) Complex,” Journal of the American Chemical Society 142 (2020): 19360, 10.1021/jacs.0c09639. 33112128

[32] F. Alonso , I. P. Beletskaya , and M. Yus , “Metal‐Mediated Reductive Hydrodehalogenation of Organic Halides,” Chemical Reviews 102 (2002): 4009, 10.1021/cr0102967. 12428984

[33] S. Bajo , E. Soto , M. Fernández‐Buenestado , J. López‐Serrano , and J. Campos , “A Low‐Coordinate Platinum(0)‐Germylene for E–H Bond Activation and Catalytic Hydrodehalogenation,” Nature Communications 15 (2024): 9656, 10.1038/s41467-024-53940-9. PMC11543666 39511155

[34] M. Claros , F. Ungeheuer , F. Franco , V. Martin‐Diaconescu , A. Casitas , and J. Lloret‐Fillol , “Reductive Cyclization of Unactivated Alkyl Chlorides With Tethered Alkenes Under Visible‐Light Photoredox Catalysis,” Angewandte Chemie International Edition 131 (2019): 4923, 10.1002/ange.v131.15. PMC6519206 30707782

[35] M. Rausch , C. Bruhn , and D. Steinborn , “ω‐Monofluoro‐alkylrhodoximes: Synthesis and Structures of [Rh {(CH_2_)_n_F}(dmgH)_2_ (PPh_3_)](n= 1, 3) and C‐F Activation Reactions,” Journal of Organometallic Chemistry 622 (2001): 172.

[36] S. Mollasalehi , T. Braun , and J. Borel , “Activation and Derivatization of Perfluoro(methyl Vinyl Ether) by a Rh(I) Germyl Complex,” European Journal of Inorganic Chemistry 29 (2025): e202500509, 10.1002/ejic.v29.5.

[37] S. Mollasalehi , M. Ahrens , and T. Braun , “Activation of Perfluoro(methyl Vinyl ether) at Rh(I) Complexes: Metal‐Centered Versus Phosphine‐Mediated Decarbonylation,” Chemical Science 16 (2025): 10928, 10.1039/D5SC02056E. PMC12086712 40395377

[38] M. Bui , F. Oesten , P. Heße , et al., “Catalytic Surface Degradation of (CF_2_H)_2_O to CF_3_H at a Si‐Teflate Doped Aluminium Chlorofluoride Lewis Superacid,” Dalton Transactions 55 (2026): 10502, 10.1039/D6DT01447J. 42417405

[39] B. J. Jelier , J. L. Howell , C. D. Montgomery , D. B. Leznoff , and C. M. Friesen , “A Convenient Route to Tetraalkylammonium Perfluoroalkoxides From Hydrofluoroethers,” Angewandte Chemie International Edition 127 (2015): 2988, 10.1002/ange.v127.10. 25612560

[40] C. Huang and L. V. Kudzma , Baxter International Inc., WO2010/123880A1, 2010.

[41] C. Huang , V. S. Venturella , A. L. Cholli , F. M. Venutolo , A. T. Silbermann , and G. G. Vernice , “Detailed Investigation of Fluoromethyl 1,1,1,3,3,3‐Hexafluoro‐2‐Propyl ether (sevoflurane) and Its Degradation Products. Part I: Synthesis of Fluorinated, Soda Lime Induced Degradation Products,” Journal of Fluorine Chemistry 45 (1989): 239, 10.1016/S0022-1139(00)84149-4.

[42] D. Noveski , T. Braun , and S. Krückemeier , “Synthesis and Reactivity of Rhodium Fluoro Complexes,” Journal of Fluorine Chemistry 125 (2004): 959, 10.1016/j.jfluchem.2004.01.024.

[43] N. Pfister , T. Braun , P. Wittwer , and M. Ahrens , “Selective Formation and Characterization of a Rh^III^ Difluorido λ^4^‐Trifluorosulfanyl Complex,” Zeitschrift Fur Anorganische Und Allgemeine Chemie 644 (2018): 1064.

[44] S. A. Macgregor , D. C. Roe , W. J. Marshall , K. M. Bloch , V. I. Bakhmutov , and V. V. Grushin , “The F/Ph Rearrangement Reaction of [(Ph_3_P)_3_RhF], the Fluoride Congener of Wilkinson’s Catalyst,” Journal of the American Chemical Society 127 (2005): 15304, 10.1021/ja054506z. 16248673

[45] V. V. Grushin and W. J. Marshall , “The Fluoro Analogue of Wilkinson’s Catalyst and Unexpected Ph−Cl Activation,” Journal of the American Chemical Society 126 (2004): 3068, 10.1021/ja049844z. 15012134

[46] S. Erhardt and S. A. Macgregor , “Computational Study of the Reaction of C_6_F_6_ With [IrMe(PEt_3_)_3_]: Identification of a Phosphine‐Assisted C−F Activation Pathway via a Metallophosphorane Intermediate,” Journal of the American Chemical Society 130 (2008): 15490, 10.1021/ja804622j. 18950169

[47] O. Blum , F. Frolow , and D. Milstein , “C–F Bond Activation by Iridium(I). A Unique Process Involving P–C Bond Cleavage, P–F Bond Formation and Net Retention of Oxidation State,” Journal of the Chemical Society, Chemical Communications 27 (1991): 258, 10.1039/C39910000258.

[48] N. A. Jasim , R. N. Perutz , A. C. Whitwood , et al., “Contrasting Reactivity of Fluoropyridines at Palladium and Platinum: C−F Oxidative Addition at Palladium, P−C and C−F Activation at Platinum,” Organometallics 23 (2004): 6140, 10.1021/om049448p.

[49] R. D. Bagnall , W. Bell , K. Pearson , A. Jeater , “New Inhalation Anaesthetics: III. Fluorinated Aliphatic Ethers,” Journal of Fluorine Chemistry 13 (1979): 123.

[50] T. Ahrens , M. Teltewskoi , M. Ahrens , T. Braun , and R. Laubenstein , “Competing Reaction Pathways of 3,3,3‐Trifluoropropene at Rhodium Hydrido, Silyl and Germyl Complexes: C‐F Bond Activation Versus Hydrogermylation,” Dalton Transactions 45 (2016): 17495, 10.1039/C6DT03027K. 27739544

[51] M. Talavera , R. Müller , T. Ahrens , et al., “Activation of Tetrafluoropropenes by Rhodium(I) Germyl and Silyl Complexes,” Faraday Discussions 220 (2019): 328, 10.1039/C9FD00059C. 31538175

[52] M. Talavera and T. Braun , “Versatile Reaction Pathways of 1,1,3,3,3‐Pentafluoropropene at Rh(I) Complexes [Rh(E)(PEt_3_)_3_] (E=H, GePh_3_, Si(OEt)_3_, F, Cl): C‐F Versus C‐H Bond Activation Steps,” Chemistry–A European Journal 27 (2021): 11926, 10.1002/chem.v27.46. PMC8456946 34118095

[53] A. Van der Ent , A. Onderdelinden , and R. A. Schunn , “Chlorobis (cyclooctene) Rhodium (I) and‐Iridium (I) Complexes,” Inorganic Syntheses 20 (1973): 92, 10.1002/SERIES2146.

[54] G. Meier and T. Braun , “Hydrogenation of a Rhodium Peroxido Complex by Formate Derivatives: Mechanistic Studies and the Catalytic Formation of H_2_O_2_ From O_2_ ,” Angewandte Chemie International Edition 124 (2012): 12732, 10.1002/ange.v124.50. 23125187

[55] C. N. von Hahmann , M. Talavera , C. Xu , and T. Braun , “Reactivity of 3,3,3‐Trifluoropropyne at Rhodium Complexes: Development of Hydroboration Reactions,” Chemistry – A European Journal 24 (2018): 11131, 10.1002/chem.v24.43. 29791749

[56] D. Milstein , “The First Isolated, Stable Cis‐Hydridoalkylrhodium Complexes and Their Reductive Elimination Reaction,” Journal of the American Chemical Society 104 (1982): 5227, 10.1021/ja00383a039.

[57] A. Foris , “ ^19^F and ^1^H NMR Spectra of Halocarbons,” Magnetic Resonance in Chemistry 42 (2004): 534. 10.1002/mrc.1368 15137047

[58] Y. Liang , Z. Zhao , and N. Shibata , “Pd‐Catalyzed Fluoro‐Carbonylation of Aryl, Vinyl, and Heteroaryl Iodides Using 2‐(difluoromethoxy)‐5‐Nitropyridine,” Communications Chemistry 3 (2020): 59, 10.1038/s42004-020-0304-3. PMC9814140 36703482

[59] M. Kolli , M. Hamidouche , N. Bouaouadja , and G. Fantozzi , “HF Etching Effect on Sandblasted Soda‐Lime Glass Properties,” Journal of the European Ceramic Society 29 (2009): 2697, 10.1016/j.jeurceramsoc.2009.03.020.

[60] T. Hanamoto , N. Morita , and K. Shindo , “Synthesis and Reactions of (2,2,2‐Trifluoroethyl)triphenylphosphonium Trifluoromethanesulfonate,” European Journal of Organic Chemistry 2003 (2003): 4279, 10.1002/ejoc.v2003:21.

[61] N. Allefeld , B. Neumann , H. G. Stammler , G. V. Röschenthaler , N. Ignat’ev , and B. Hoge , “Synthesis and Reactivity of New Functionalized Perfluoroalkylfluorophosphates,” Chemistry – A European Journal 20 (2014): 7736, 10.1002/chem.v20.25. 24827420

[62] L. Hartmann , K. Wels , N. Tiessen , et al., “Bis (trifluoromethyl) Disulfide and the EtP_4_ Phosphazene Base—Formation of a Bench‐Stable [EtP_4_SCF_3_]^+^[SCF_3_]^−^ Salt,” Chemistry – A European Journal 32 (2026): e70811, 10.1002/chem.v32.18. PMC13174922 41736704

[63] A. A. Peterson , K. A. Thoreson , and K. McNeill , “Phosphinorhodium‐Catalyzed Dehalogenation of Chlorinated and Fluorinated Ethylenes: Distinct Mechanisms With Triethylsilane and Dihydrogen,” Organometallics 28 (2009): 5982, 10.1021/om900718s.

[64] A. A. Peterson and K. McNeill , “Catalytic Dehalogenation of sp^2^ C−F and C−Cl Bonds in Fluoro‐and Chloroalkenes,” Organometallics 25 (2006): 4938, 10.1021/om0607292.

