# Supplementary material for: Reactivity of Rh(I) Complexes Toward Fluorinated Ethers: Activation of Inhalation Anesthetics
Source: ChemistryOpen. 2026 Nov 1;15(11):e70328. doi: 10.1002/open.70328 (PMC13724305; doi:10.1002/open.70328)
Supplement: Supplementary file 1 [file OPEN-15-e70328-s001.pdf]

Supporting Information

Reactivity of Rh(I) Complexes towards Fluorinated Ethers: Activation of Inhalation Anesthetics

Soodeh Mollasalehi,[a] Jakob Bölle,[a] Thomas Braun*[a]

[a] S. Mollasalehi, J. Bölle, Prof. Dr. T. Braun
Department of Chemistry
Humboldt-Universität zu Berlin
Brook-Taylor-Straße 2, 12489 Berlin (Germany)
E-mail: thomas.braun@cms.hu-berlin.de

Table of Contents

1 Synthesis and characterization of all compounds 3
1.1 General Procedures, Methods and Materials 3
1.2 Reaction of [Rh(H)(PEt_3)$_3$] (**1**) with sevoflurane 3
1.3 Reaction of [Rh(H)(PEt_3)$_3$] (**1**) with enflurane 4
1.4 Reaction of [Rh(H)(PEt_3)$_3$] (**1**) with isoflurane 5
2 NMR Spectra 6
3 Kinetic Study 17
4 References 19

1 Synthesis and characterization of all compounds

1.1 General Procedures, Methods and Materials

All samples were prepared in a glovebox and the reactions performed using conventional Schlenk techniques. Solvents were dried by conventional methods[1] and, prior to use, distilled under argon. All other reagents were obtained from commercial sources. Solids were dried in high vacuum, and liquids were degassed and stored over activated molecular sieves prior to usage. The NMR spectroscopic measurements were recorded either on a Bruker Avance 300, a Bruker Avance III 300, or a Bruker Avance 600 spectrometer. ^{1}H and ^{13}C{^{1}H} signals are referred to residual solvent signals, those of ^{31}P{^{1}H} to 85% H_3PO_4 and the ^{19}F NMR spectra to external $CFCl_3$. ^{1}H, ^{19}F and ^{31}P{^{1}H} signal assignments were confirmed by ^{1}H{^{31}P}, ^{1}H{^{19}F}, ^{19}F{^{1}H}, ^{1}H,^{1}H COSY, ^{19}F,^{19}F COSY, ^{1}H,^{31}P HMBC, ^{1}H,^{19}F COSY experiments. The rhodium complex [Rh(H)(PEt_3)$_3$] (**1**) was prepared as described in the literature.[2] For quantitative analyses, α,α,α-trifluorotoluene or Tris(4-fluorophenyl)phosphine oxide were added as internal standard and the parameter for recording the ^{19}F NMR spectra were adjusted (D1 = 30 s, AQ = 2 s).

1.2 Reaction of [Rh(H)(PEt_3)$_3$] (1) with sevoflurane

In a Young NMR tube [Rh(H)(PEt_3)$_3$] (**1**) (40 mg, 0.087 mmol) was dissolved in C_6D_6 (0.4 mL) and sevoflurane (17 µl, 1.31 mmol) was added to the solution. The tube was warmed to 328K. The reaction was monitored by NMR spectroscopy. After 36 hours, the ^{31}P{^{1}H}, ^{1}H and ^{19}F NMR spectroscopic data revealed the full conversion of **1** into [Rh(F)(PEt_3)$_3$] (**2**), *trans*-[Rh(F)(CO)(PEt_3)$_2$] (**3**), and *cis-fac*-[Rh(F)(H)$_2$(PEt_3)$_3$] (**4**) in a ratio of 1:0.3:0.2. Additionally, the formation of $(CF_3)_2CHOCH_3$ was observed. When the tube was heated for a longer time, the evolution of **2** and **4** into **3**, *trans*-[Rh(F)(Et_2PF)(PEt_3)$_2$] (**5**) as well as ethane was observed (**2**:**3**:**4**:**5** ratio is 1:0.8:0:1 after 4 days). Complex **5** was characterised by ^{31}P{^{1}H}, ^{1}H and ^{19}F NMR spectroscopy.

Analytic data for *trans*-[Rh(F)(Et_2PF)(PEt_3)$_2$] (5): ^{1}H NMR (300.1 MHz, C_6D_6): δ = 2.07-1.43 (m, P*CH$_2$*CH$_3$), 1.41-0.64 (m, PCH$_2$*CH$_3$*) ppm, resonances corresponding to the phosphine ligands are partially overlapping. **^{31}P{^{1}H} NMR** (121.5 MHz, C_6D_6): δ = 220.4 (dddt, $^{1}J_{P,F}$= 818.3 Hz, $^{2}J_{P,F}$ = 216.2 Hz, $^{1}J_{P,Rh}$= 208.0, $^{2}J_{P,P}$= 41.6 Hz, 1P), 25.8 (ddt, $^{1}J_{P,Rh}$ = 136.1 Hz, $^{2}J_{P,P}$ = 41.6 Hz, $^{3}J_{P,F}$~ $^{3}J_{P,F}$ = 26.2 Hz, 1P) ppm. **^{19}F NMR** (282.4 MHz, C_6D_6): δ = –132.3 (dm, $^{1}J_{F,P}$ = 818; ddddd in ^{19}F{^{1}H} NMR spectrum: $^{1}J_{F,P}$ = 818, $^{2}J_{F,P}$ = 26, $^{2}J_{F,Rh}$ = 20, $^{3}J_{F,F}$ = 12 Hz, 1F, P*F*), –261.1 (ddtd, $^{2}J_{F,P}$ = 216 Hz, $^{2}J_{F,Rh}$ = 42 Hz, $^{2}J_{F,P}$ = 26, $^{3}J_{F,F}$ = 12 Hz, Rh*F*) ppm.

1.3 Reaction of [Rh(H)(PEt_3)$_3$] (**1**) with enflurane

In a Young NMR tube [Rh(H)(PEt_3)$_3$] (**1**) (43 mg, 0.094 mmol) was dissolved in C_6D_6 (0.4 mL) and enflurane (33 µl, 0.27 mmol) was added to the solution. The reaction was monitored by NMR spectroscopy at room temperature. After 5 minutes, the $^{31}P\{^1H\}$, ^1H and ^{19}F NMR spectroscopic data revealed the full conversion of **1** into [Rh(H)(Cl)($CFHCF_2OCF_2H$)(PEt_3)$_3$] (**8**). The reaction was monitored for 1 day, which showed the evolution of **8** into **7** and $CFH_2CF_2OCF_2H$[3]. Complex **8** was characterised by $^{31}P\{^1H\}$, ^1H and ^{19}F NMR spectroscopy.

Analytic data for [Rh(H)(Cl)($CFHCF_2OCF_2H$)(PEt_3)$_3$] (8): ^1H NMR (600 MHz, 253K tol-d_8): δ = 6.2 (t, $^2J_{H,Fe}$ ~ $^2J_{H,Fd}$ = 71.9 Hz, s in $^1H\{^{19}F\}$, CF_2H^c), 5.9 (dm, $^2J_{H,Fa}$ = 46.5 Hz, 1H, CFH^b), 2.29-1.40 (m, $P^aCH_2CH_3$), 1.86-1.55 (m, $P^bCH_2CH_3$), 1.14-0.91 (m, $P^aCH_2CH_3$), 1.03-0.91 (m, $P^bCH_2CH_3$), resonances corresponding to the phosphine ligands are partially overlapping; The phosphorus-proton correlation was confirmed by ^1H-^{31}P HMBC NMR spectrum, –10.5 (dq, $^2J_{H,P}$ = 183.4, $^2J_{H,P}$ ~ $^1J_{H,Rh}$ = 14.4 Hz, d in $^1H\{^{31}P\}$, 1H, RhH^a) ppm. **$^{31}P\{^1H\}$ NMR** (121.5 MHz, C_6D_6): δ = 16.3 (ddm, $^1J_{P,Rh}$= 103.0 Hz, $^2J_{P,Pb}$= 22.8 Hz, 2P, P^a), –8.3 (dtd, $^1J_{P,Rh}$ = 85.7 Hz, $^3J_{P,Fa}$= 42.6 Hz, $^2J_{P,Pa}$ = 22.8 Hz, 1P, P^b) ppm. **^{19}F NMR** (282.4 MHz, C_6D_6): δ = –71.9 (dm, ddt in $^{19}F\{^1H\}$ NMR spectrum, $^2J_{F,Fc}$ = 145 Hz, $^3J_{F,Fa}$ = 17 Hz; $^4J_{F,Fe}$ ~ $^4J_{F,Fd}$ = 5 Hz, 1F, F^b), –74.0 (dm, dddd in $^{19}F\{^1H\}$ NMR spectrum, $^2J_{F,Fb}$ = 145 Hz, $^3J_{F,Fa}$ = 15 Hz, $^4J_{F,Fd}$ = 5, $^4J_{F,Fe}$ = 3 Hz, 1F, F^c), –83.2 (ddt, $^2J_{F,Fd}$ = 175 Hz, , $^2J_{F,Hc}$ = 71 Hz, $^4J_{F,Fb}$ ~ $^4J_{F,Fc}$ = 5 Hz dt in $^{19}F\{^1H\}$ NMR spectrum; 1F, F^e), –84.3 (dddd, $^2J_{F,Fe}$ = 175 Hz, $^2J_{F,Hc}$ = 72 Hz, $^4J_{F,Fb}$ = 5, $^4J_{F,Fc}$ = 3 Hz, ddd in $^{19}F\{^1H\}$ NMR spectrum; 1F, F^d), –171.7 (m; 1F, F^a) ppm.

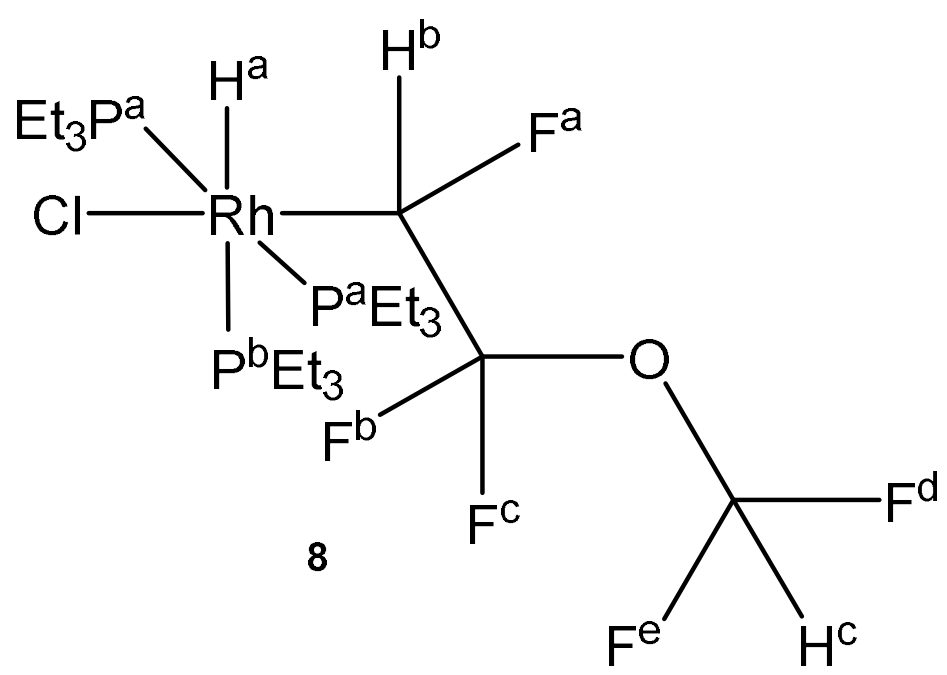

8

1.4 Reaction of [Rh(H)(PEt_3)$_3$] (**1**) with isoflurane

In a Young NMR tube [Rh(H)(PEt_3)$_3$] (**1**) (40 mg, 0.087 mmol) was dissolved in C_6D_6 (0.4 mL) and isoflurane (13 µl, 0.10 mmol) was added to the solution. The reaction was monitored by NMR spectroscopy at room temperature. After 1 hour, the $^{31}P\{^1H\}$, ^1H and ^{19}F NMR spectroscopic data revealed the full conversion of **1** into [Rh(Cl)(PEt_3)$_3$] (**7**) and the hydrofluoroether $CF_3CH_2OCF_2H$. The ether was identified as described in the literature.[4] Formation of a phosphonium salt [$CF_3CH_2PEt_3$]$_2$[SiF_6] (**9**) together with the Vaska-type complex *trans*-[Rh(Cl)(CO)(PEt_3)$_2$] (**10**) as minor side products was observed (ether/**9** ratio of 10:1 after 1 hour).

Analytic data for [$CF_3CH_2PEt_3$]$_2$[SiF_6] (9): ^{1}H NMR (300.1 MHz, C_6D_6): δ = 3.75 (dq, $^2J_{H,P}$ = 14.2, $^3J_{H,F}$ = 11.5 Hz, q in $^1H\{^{31}P\}$ NMR spectrum; CH_2), 2.47-1.43 (m, P$CH_2$$CH_3$), 1.20-0.87 (m, P$CH_2$$CH_3$) ppm, resonances corresponding to the phosphine groups are overlapping. **$^{31}P\{^1H\}$ NMR** (121.5 MHz, C_6D_6): δ = 37.0 (q, $^3J_{P,F}$= 6.2 Hz, 1P) ppm.**^{19}F NMR** (282.4 MHz, C_6D_6): δ = –55.5 (td, $^3J_{F,P}$ = 11, $^3J_{F,H}$ = 6, d in $^{19}F\{^1H\}$ NMR spectrum; CF_3), –125.9 (br s, SiF_6^{2-}) ppm.

Catalytic hydrodechlorination of isoflurane with $HSiPh_3$ and [Rh(H)(PEt_3)$_3$] (1)

In a Young NMR tube [Rh(H)(PEt_3)$_3$] (**1**) (8 mg, 0.017 mmol) and $HSiPh_3$ (114 mg, 0.44 mmol) were dissolved in C_6D_6 (0.4 mL) and isoflurane (22 µl, 0.174 mmol) was added to the solution. The reaction tube was kept in at 58 °C in oil bath and the reaction was monitored by NMR spectroscopy. After 12 days, the ^1H and ^{19}F NMR spectroscopic data revealed 75% conversion of the substrate into the hydrodechlorination product $CF_3CH_2OCF_2H$. Upon extending the reaction time to 18 days, the conversion increased to 90%.

2 NMR Spectra

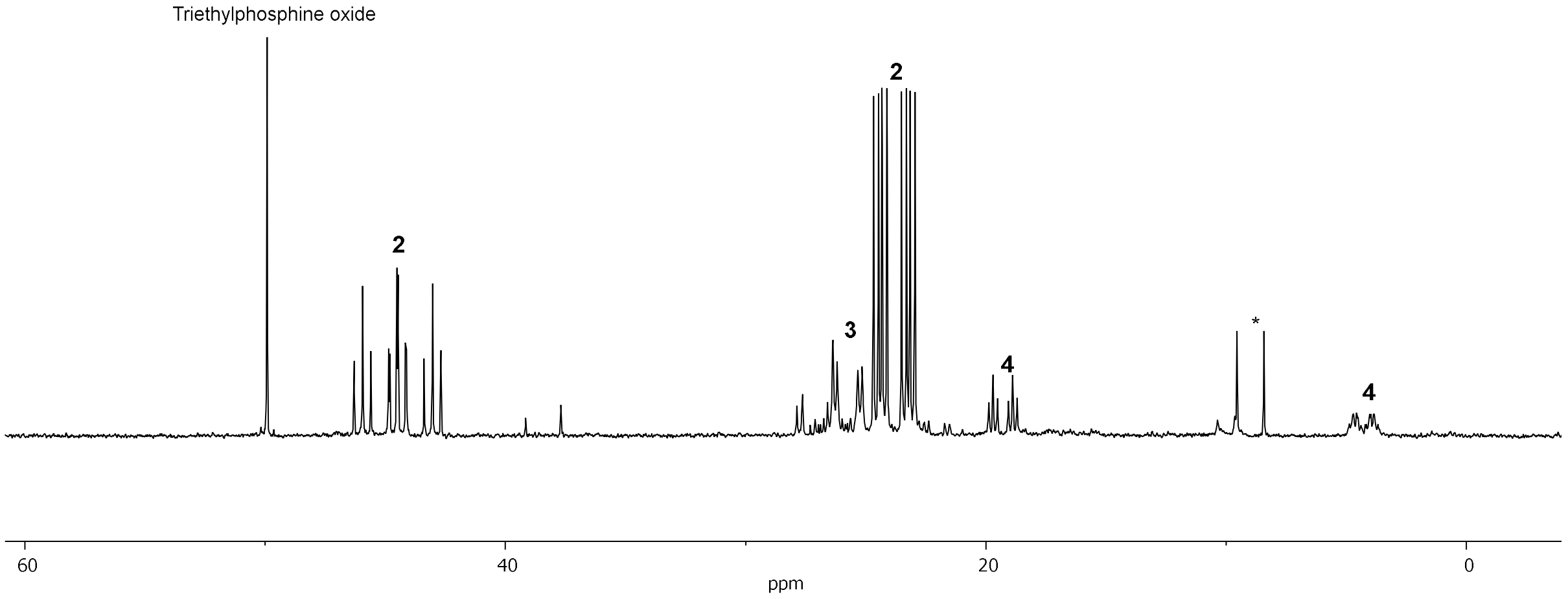

S1. $^{31}P\{^{1}H\}$ NMR spectrum of the reaction of [Rh(H)(PEt$_3$)$_3$] (**1**) with an excess of sevoflurane after 36 hours at 55 °C displaying signals for [Rh(F)(PEt$_3$)$_3$] (**2**), [Rh(F)(CO)(PEt$_3$)$_2$] (**3**), [Rh(F)(H)$_2$(PEt$_3$)$_3$] (**4**), and [Rh(PEt$_3$)$_4$][SiF$_6$] (*).

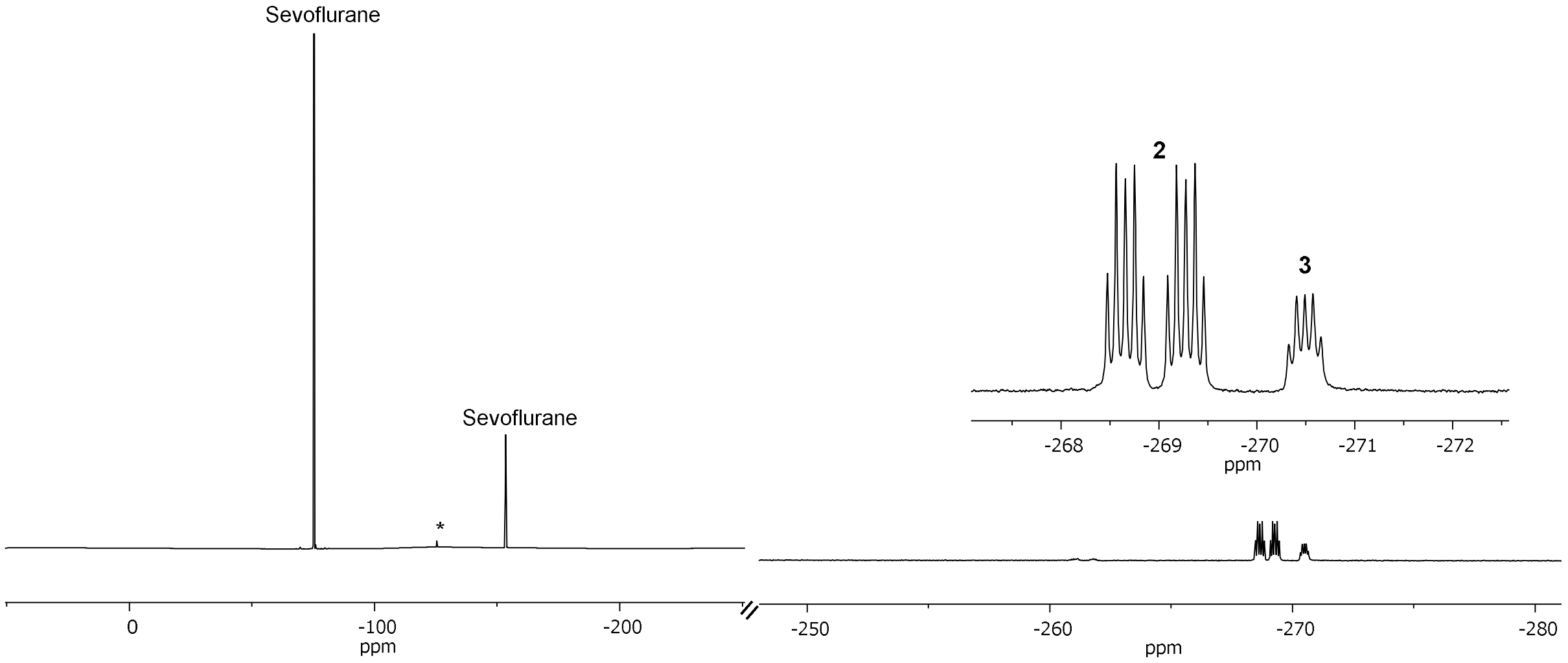

S2. ^{19}F NMR spectrum of the reaction of [Rh(H)(PEt$_3$)$_3$] (**1**) with an excess of sevoflurane in C_6D_6 after 36 hours at 55 °C displaying signals for [Rh(F)(PEt$_3$)$_3$] (**2**), *trans*-[Rh(F)(CO)(PEt$_3$)$_2$] (**3**), [Rh(F)(H)$_2$(PEt$_3$)$_3$] (**4**) (overlapping with the fluoride signal of **2**), and [Rh(PEt$_3$)$_4$][SiF$_6$] (*).

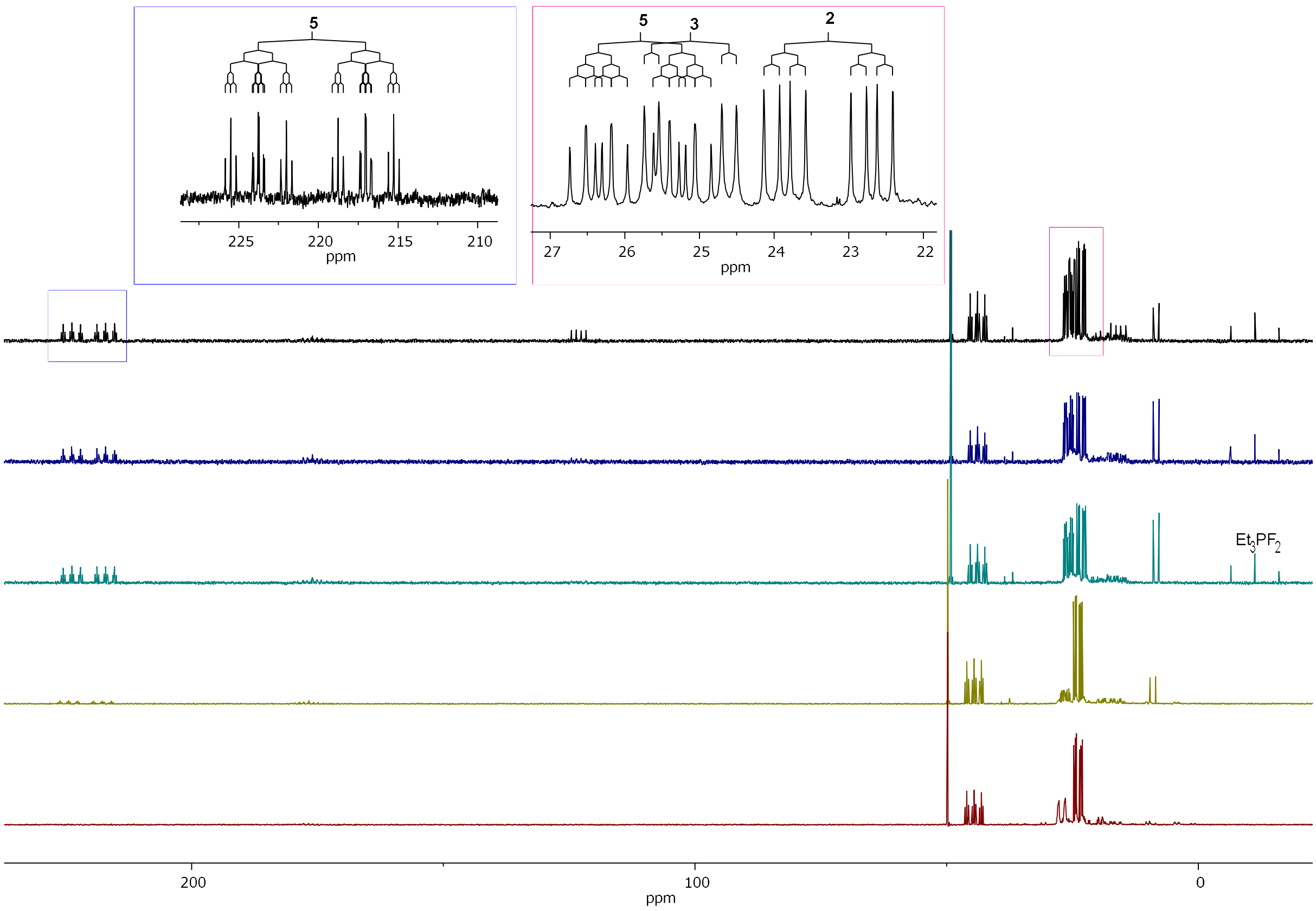

S3. $^{31}P\{^{1}H\}$ NMR spectrum of the reaction of $[Rh(H)(PEt_3)_3]$ (**1**) with an excess of sevoflurane in C_6D_6 at different time showing the conversion of complex **2** and **4** to **3** and **5**. The inset shows signals for complex **5**.

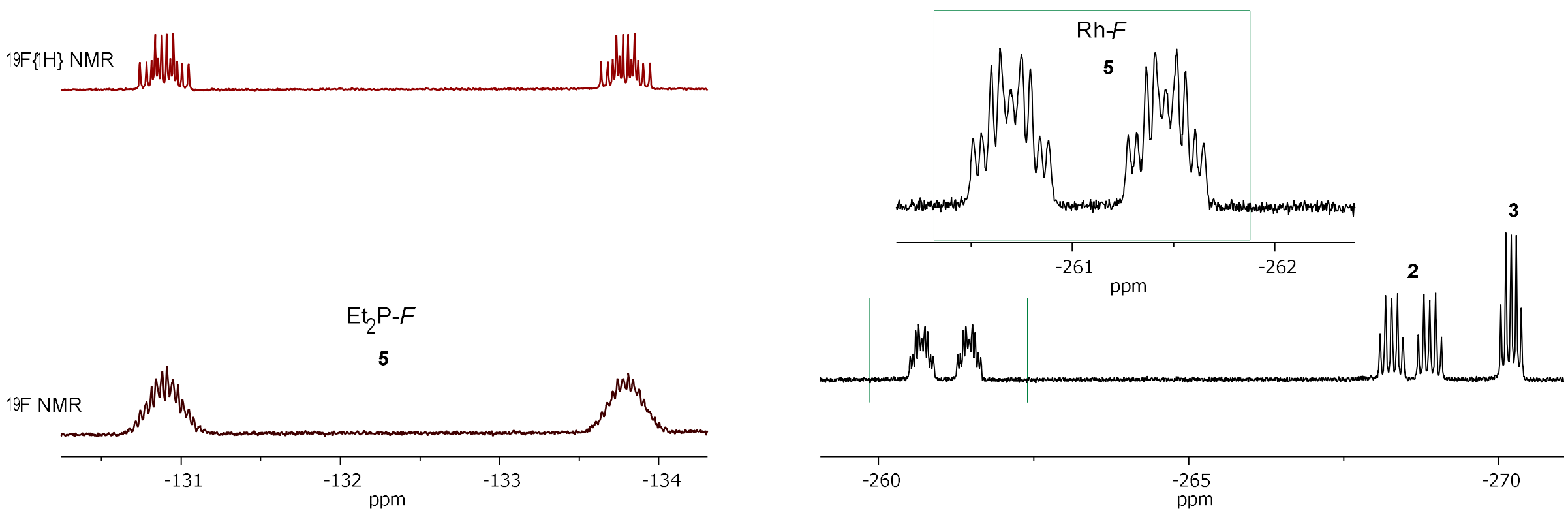

S4. Sections of ^{19}F NMR spectrum of the reaction of $[Rh(H)(PEt_3)_3]$ (**1**) with an excess of sevoflurane in C_6D_6 showing signals of **5**.

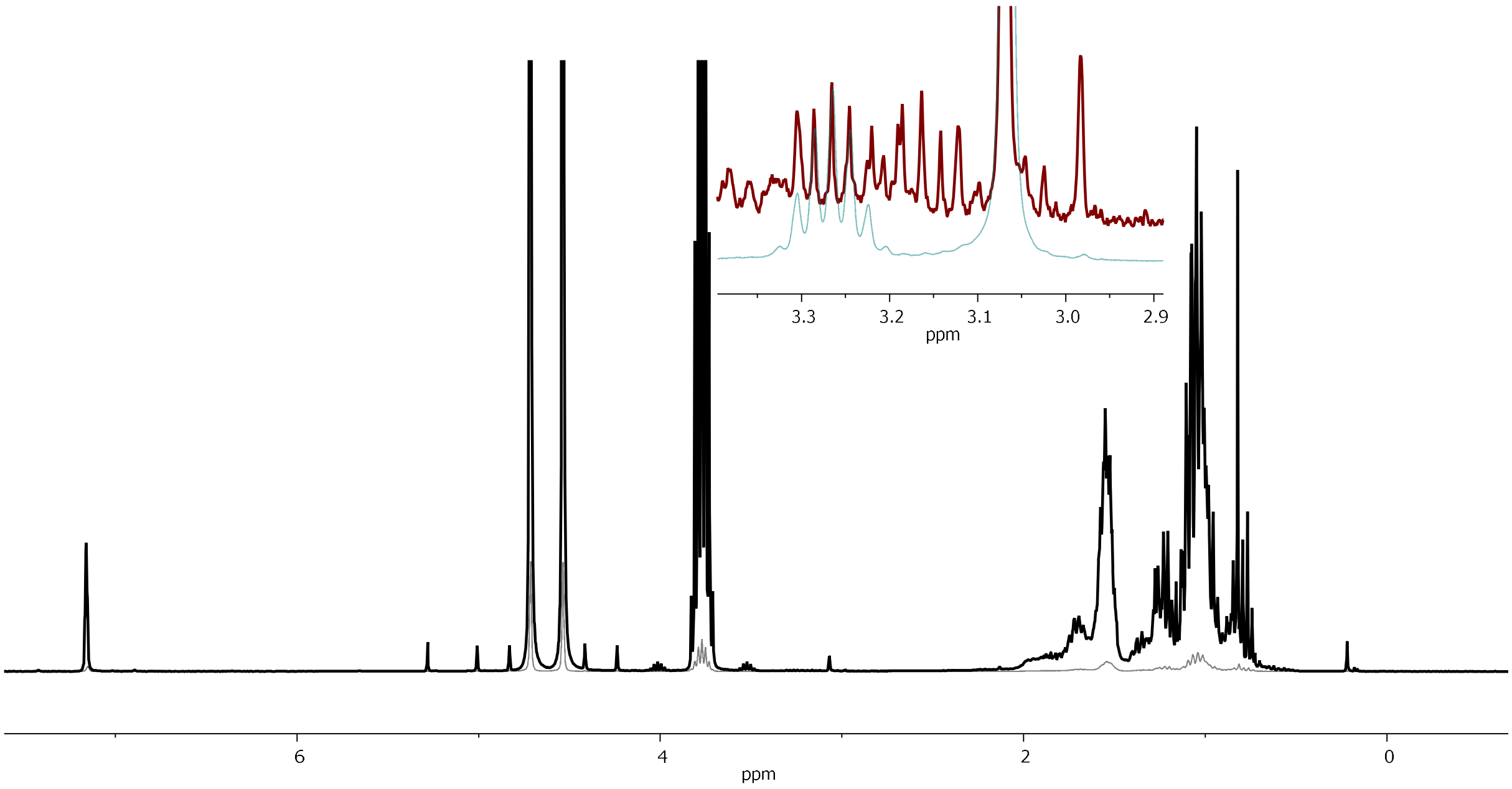

S5. ^1H NMR spectrum of the reaction of $[Rh(H)(PEt_3)_3]$ (**1**) with an excess of sevoflurane in C_6D_6 showing signals of $(CF_3)_2CHOCH_3$: Inset (red spectrum) for comparison with an internal addition of the ether to the tube (green spectrum).

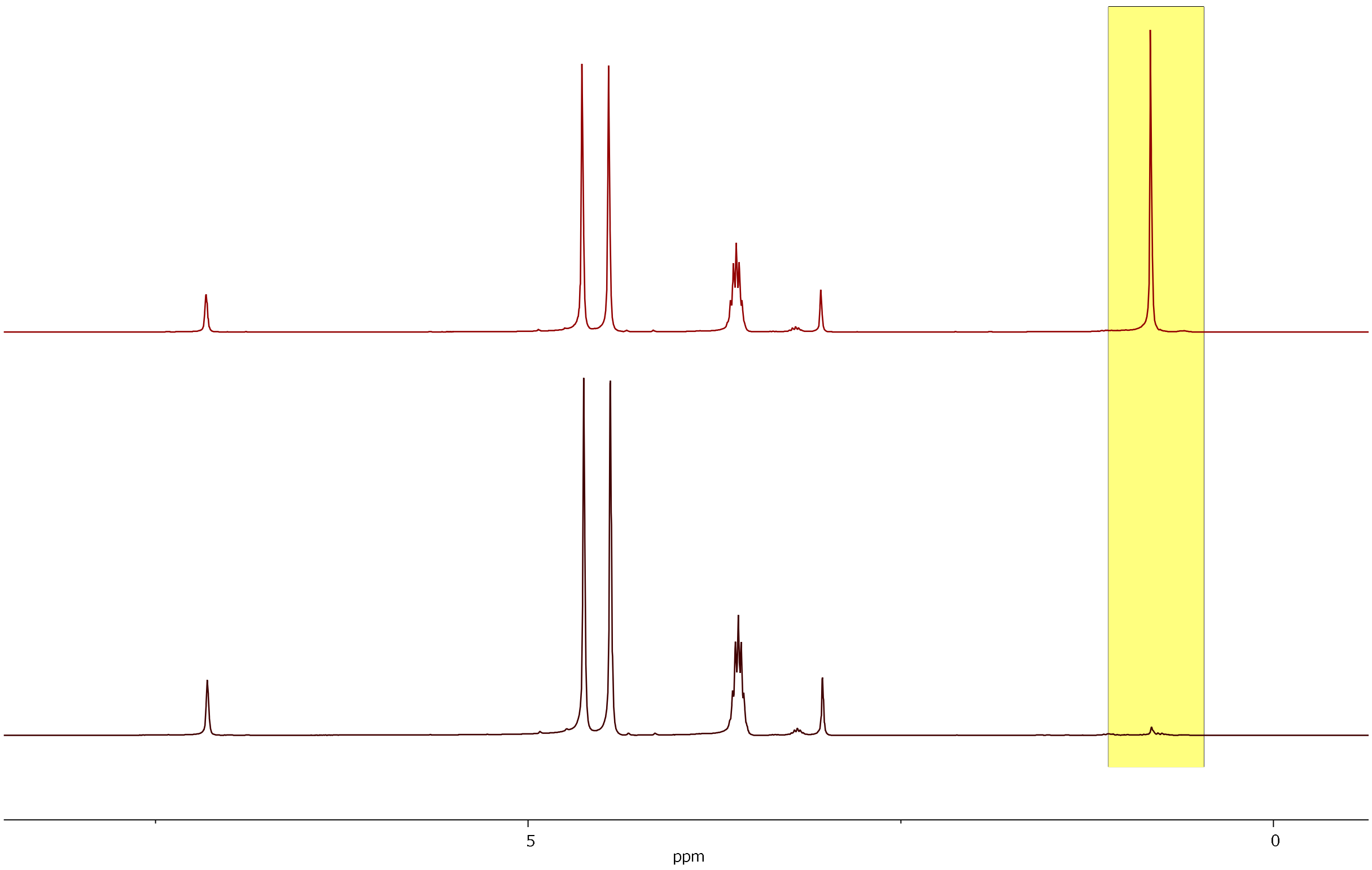

S6. ^1H NMR spectrum of the reaction of $[Rh(H)(PEt_3)_3]$ (**1**) with an excess of sevoflurane in C_6D_6 after the separation of the organic phase, showing signals for ethane (below). Addition of ethane gas to the tube (above) confirmed this observation.

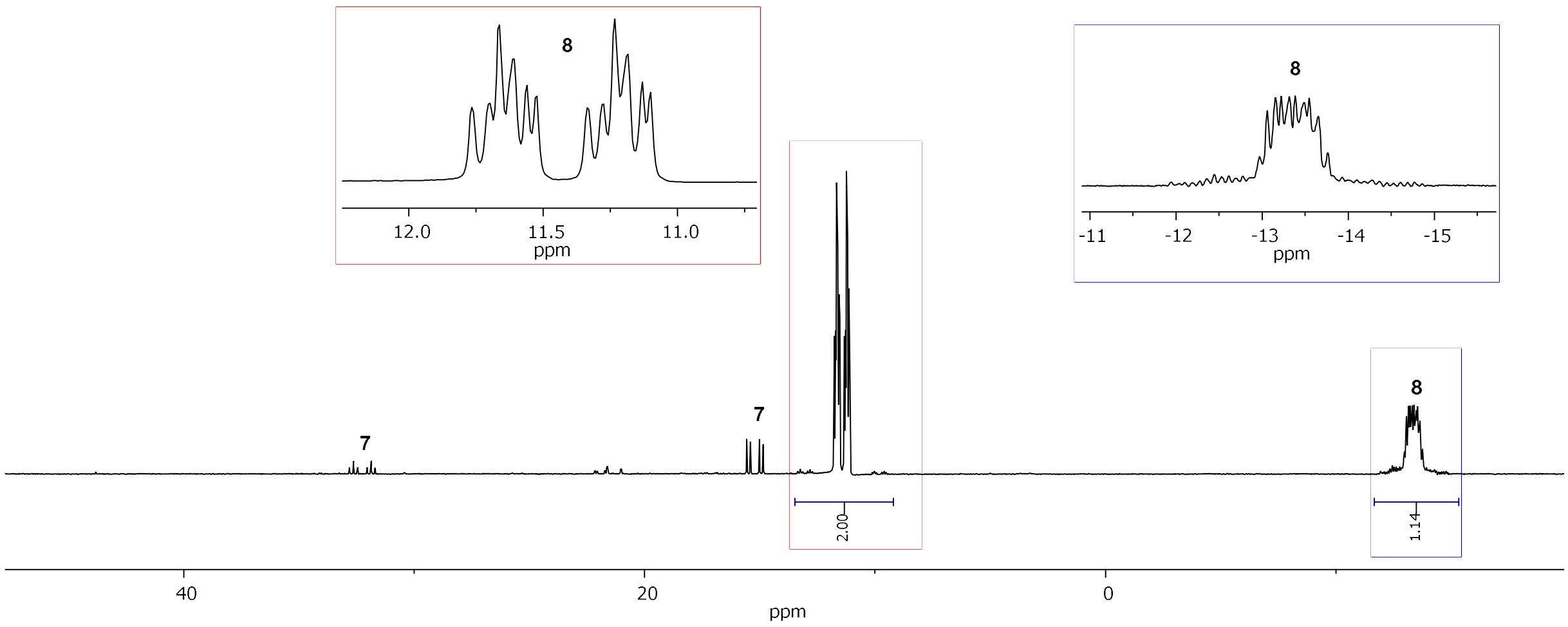

S7. $^{31}P\{^{1}H\}$ NMR spectrum (243 MHz) of the reaction of $[Rh(H)(PEt_3)_3]$ (**1**) with an excess of enflurane in tol-d_8 after 30 minutes at –40 °C signals for complex **8**.

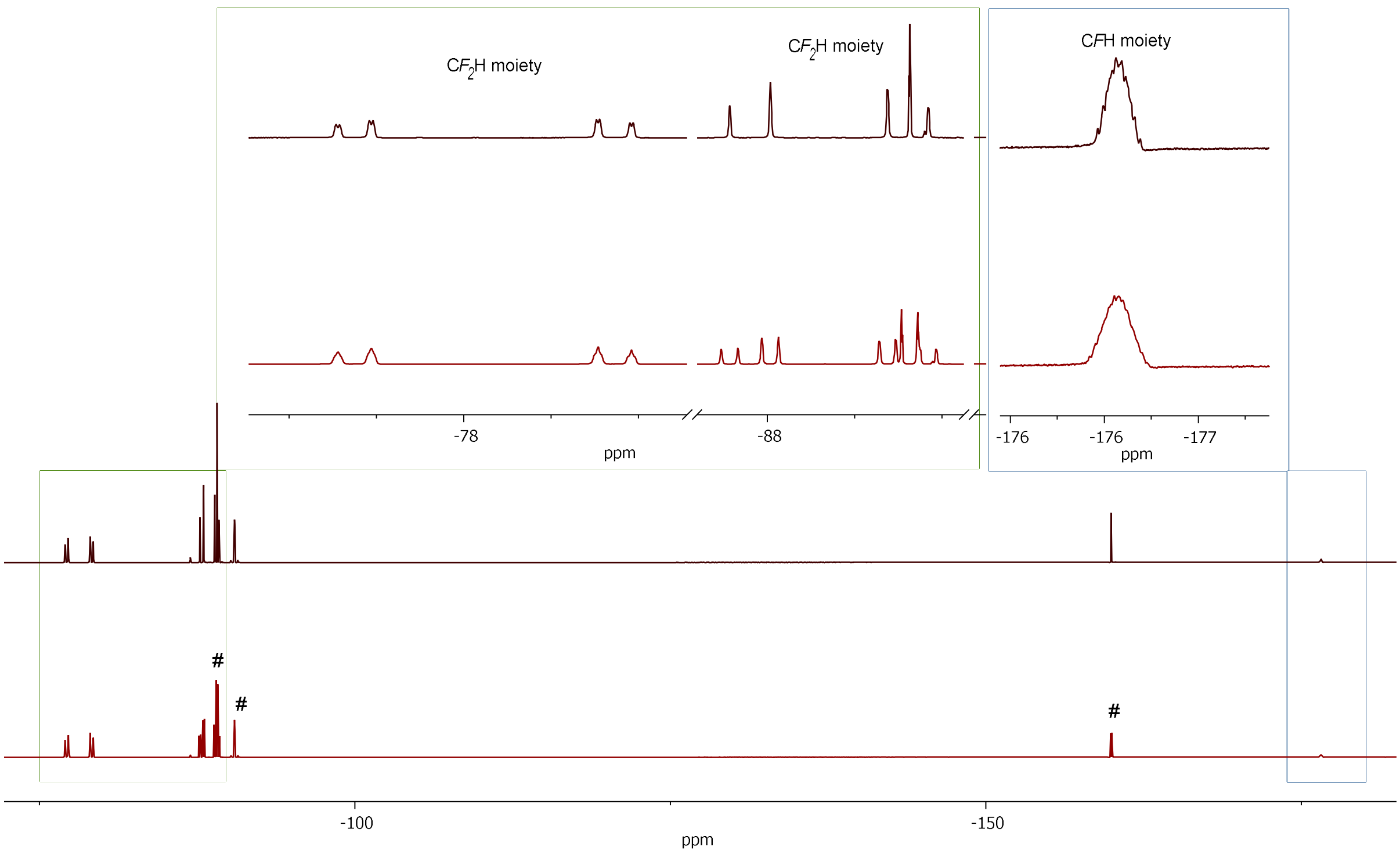

S8. ^{19}F NMR spectrum (243 MHz) of the reaction of $[Rh(H)(PEt_3)_3]$ (**1**) with an excess of enflurane in tol-d_8 after 30 minutes at –40 °C showing signals for complex **8**.

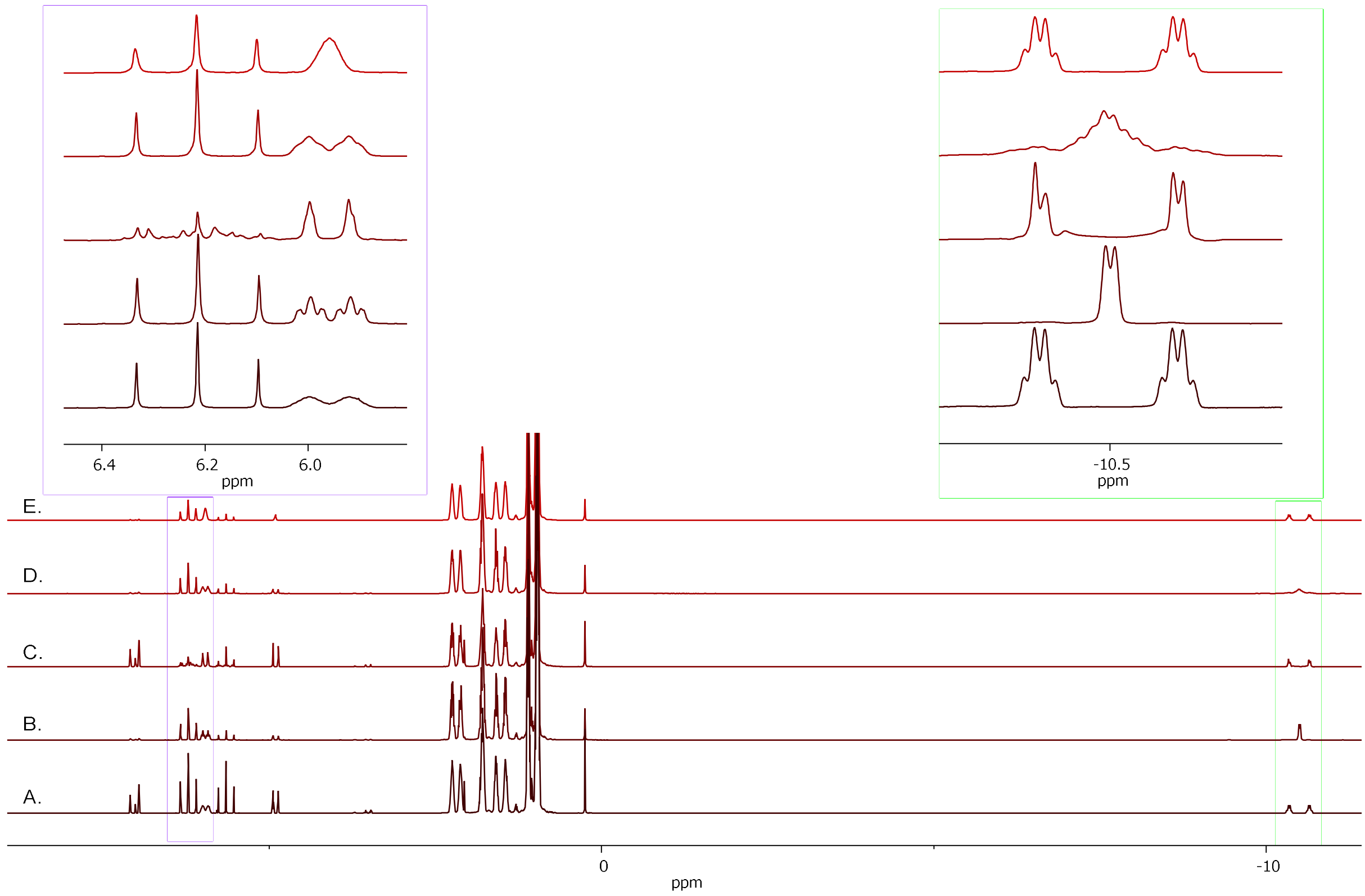

S9. ^{1}H NMR spectrum of the reaction of [Rh(H)(PEt_3)$_3$] (**1**) with an excess of enflurane (2.9 eq) in C_6D_6 after 30 minutes room temperature signals of complex **8**: **A.** ^{1}H NMR spectrum; **B.** $^{1}H\{^{31}P\}$ NMR spectrum (decoupling pulse was set at 10 ppm); **C.** $^{1}H\{^{19}F,^{31}P\}$ NMR spectrum (decoupling pulses were set at -80 ppm and 10 ppm); **D.** $^{1}H\{^{31}P\}$ NMR spectrum (decoupling pulse was set at –11 ppm.); **E.** $^{1}H\{^{19}F\}$ NMR spectrum (decoupling pulse was set at –170 ppm).

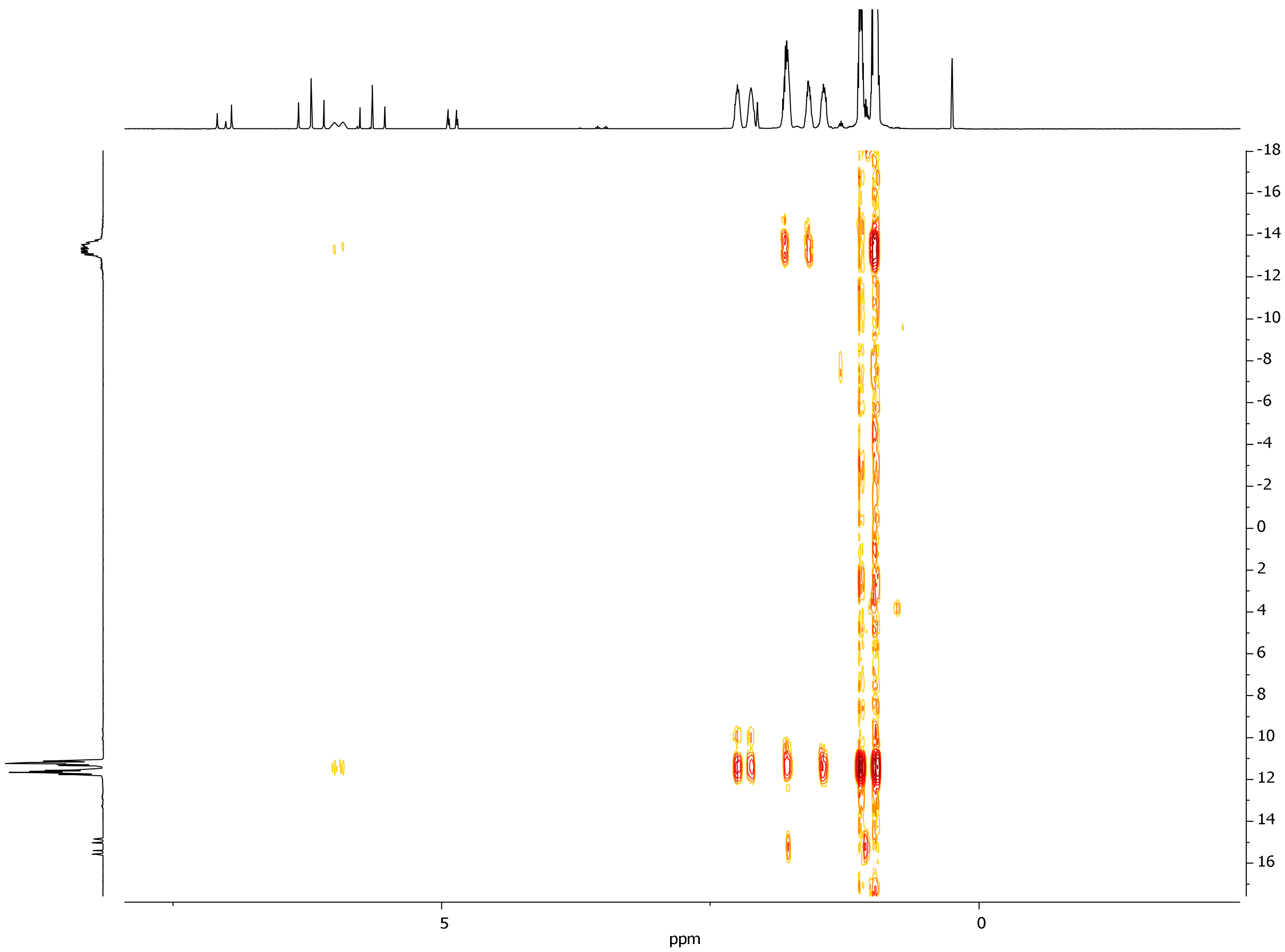

S10. ^{1}H-^{31}P HMBC NMR spectrum (600.1 - 242.9 MHz) of the reaction of [Rh(H)(PEt_3)$_3$] (**1**) with an excess of enflurane in tol-d_8 after 1 hour at –40 °C showing signals of complex **8**.

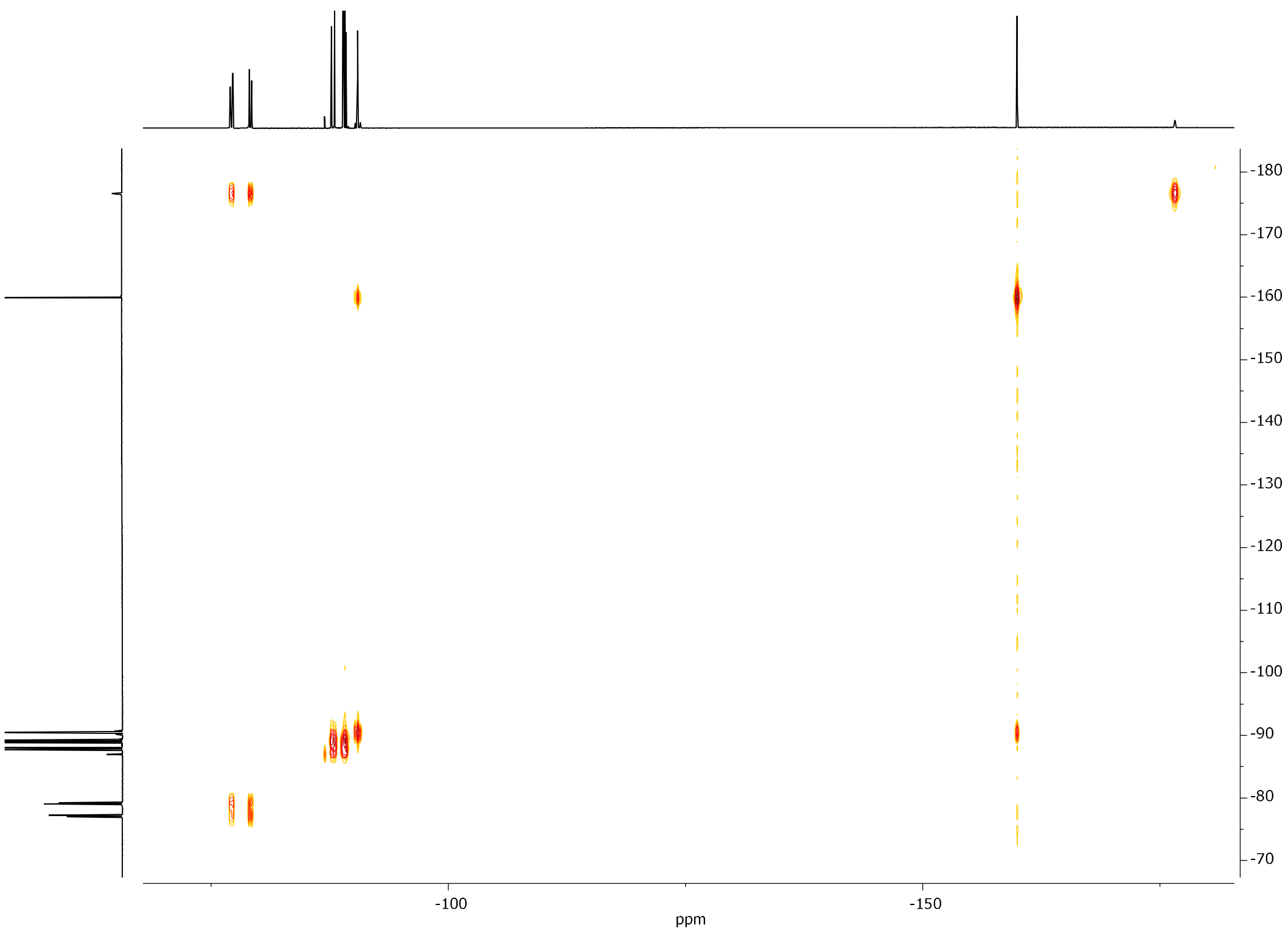

S10. ^{19}F-^{19}F COSY NMR spectrum (564.6 MHz) of the reaction of [Rh(H)(PEt$_3$)$_3$] (**1**) with an excess of enflurane in tol-d$_8$ after 1 hour at –40 °C showing signals of complex **8**.

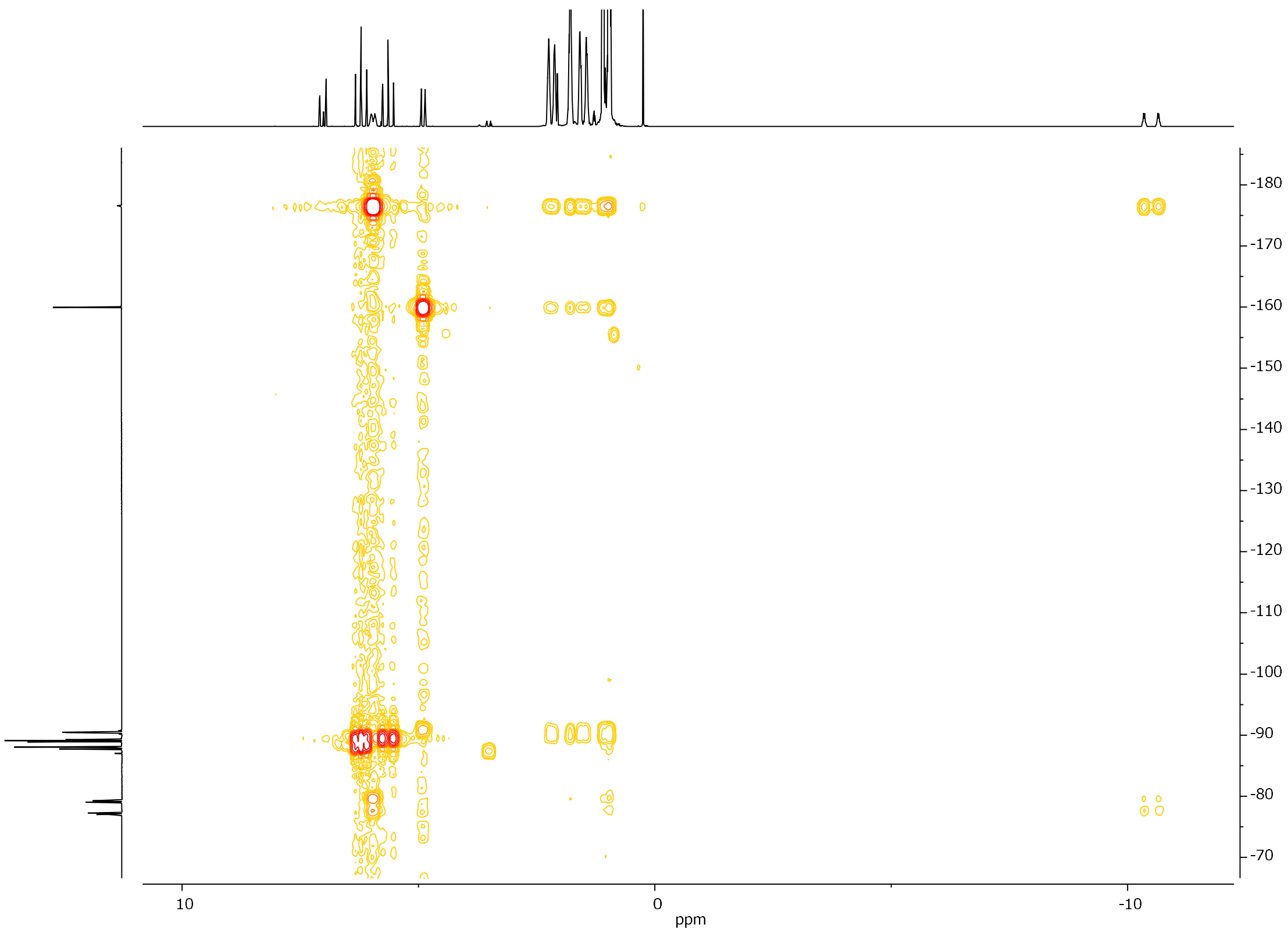

S11. ^{19}F-^{1}H COSY NMR spectrum (564.6 MHz) of the reaction of [Rh(H)(PEt$_3$)$_3$] (**1**) with an excess of enflurane in tol-d$_8$ after 1 hour at –40 °C showing signals of complex **8**.

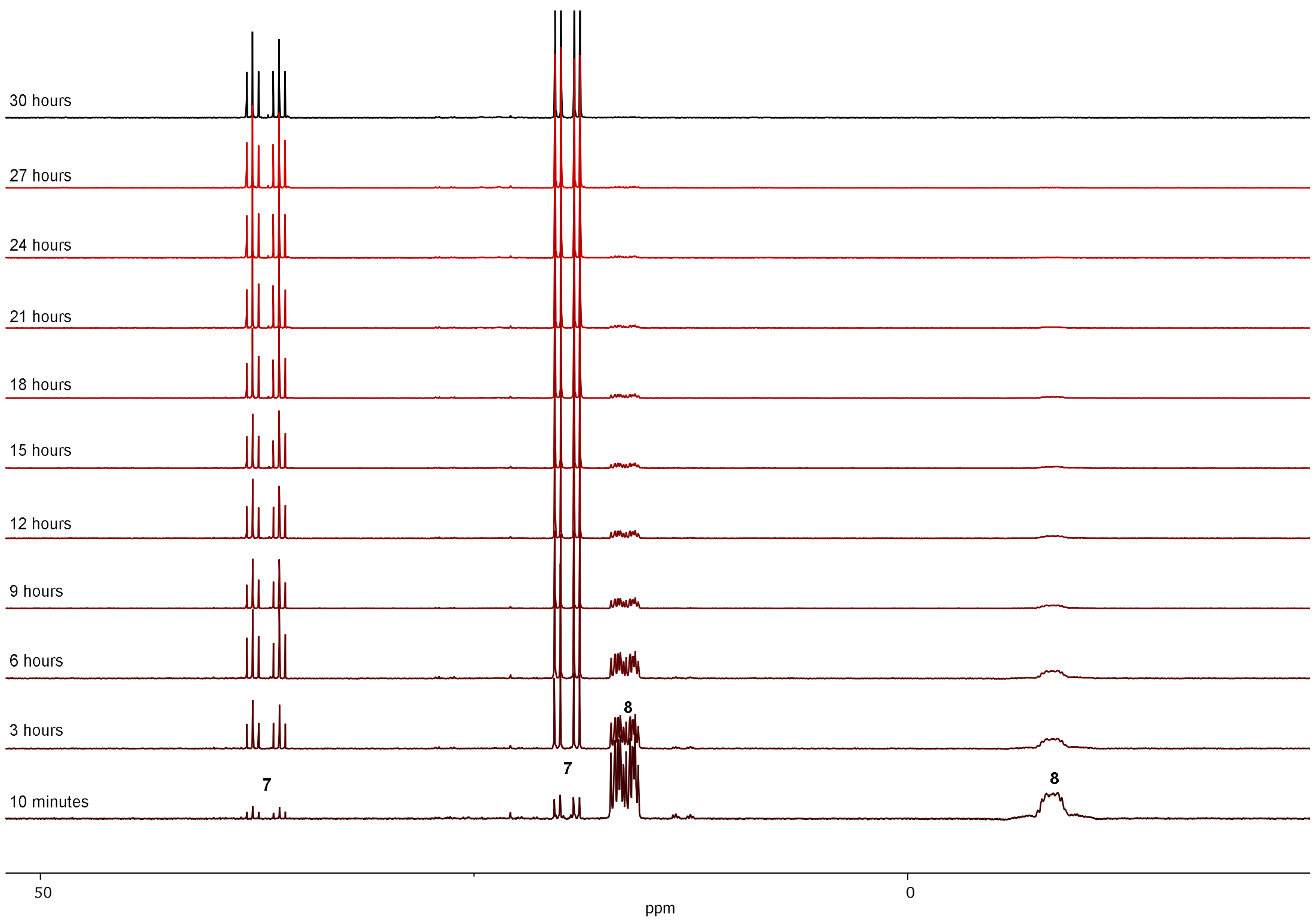

S12. $^{31}P\{^{1}H\}$ NMR spectra of the reaction of $[Rh(H)(PEt_3)_3]$ (**1**) with an excess of enflurane (2.9 eq.) in C_6D_6 at room temperature at different times showing the conversion of complex **8** to complex **7**.

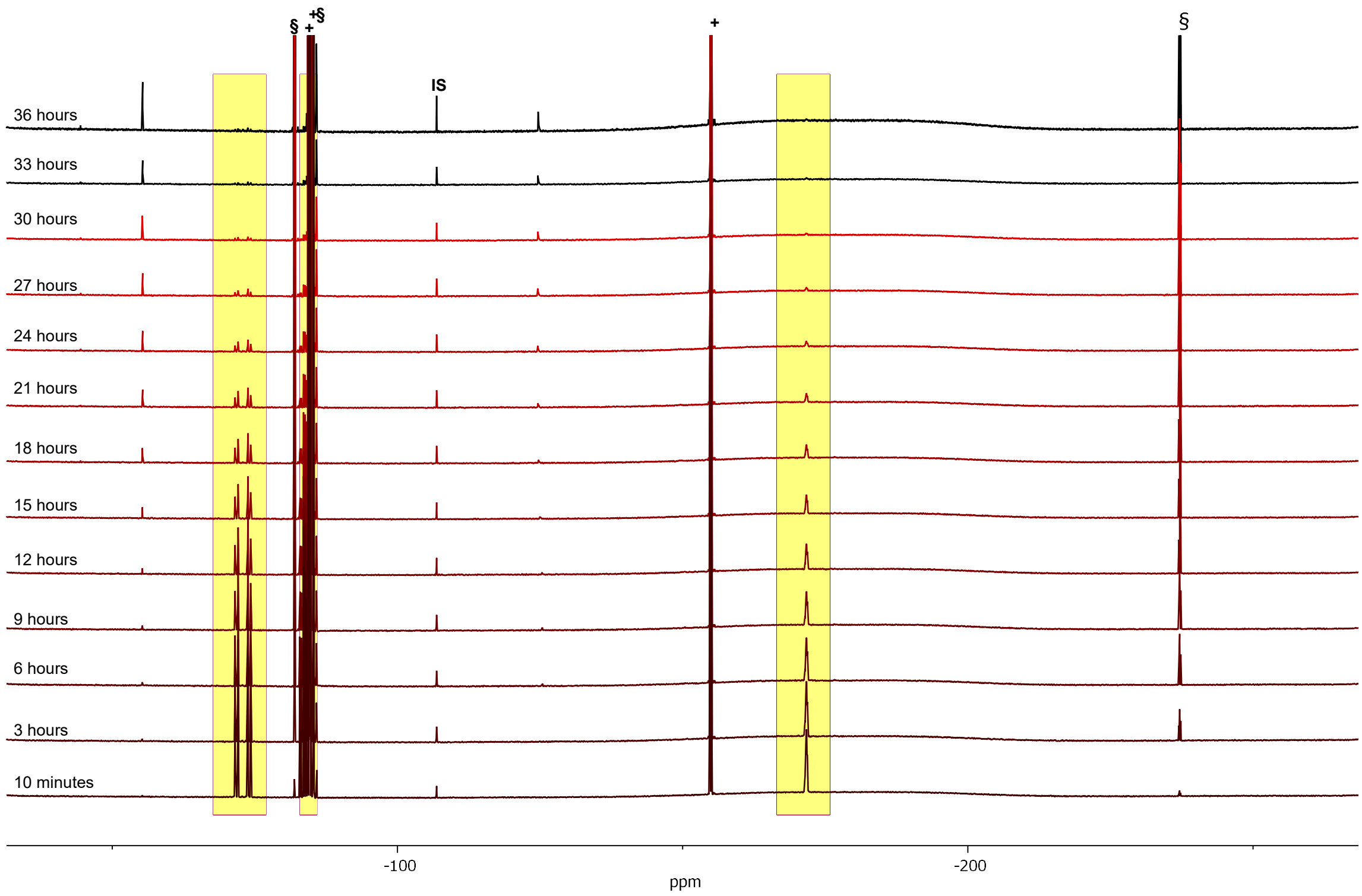

S13. ^{19}F NMR spectra of the reaction of $[Rh(H)(PEt_3)_3]$ (**1**) with an excess of enflurane (2.9 eq) (+) in C_6D_6 over time at room temperature showing the conversion of **8** (highlighted in yellow) into $CFH_2CF_2OCF_2H$ (§). Tris (4-fluorophenyl)phosphine oxide (IS) is used as an internal standard.

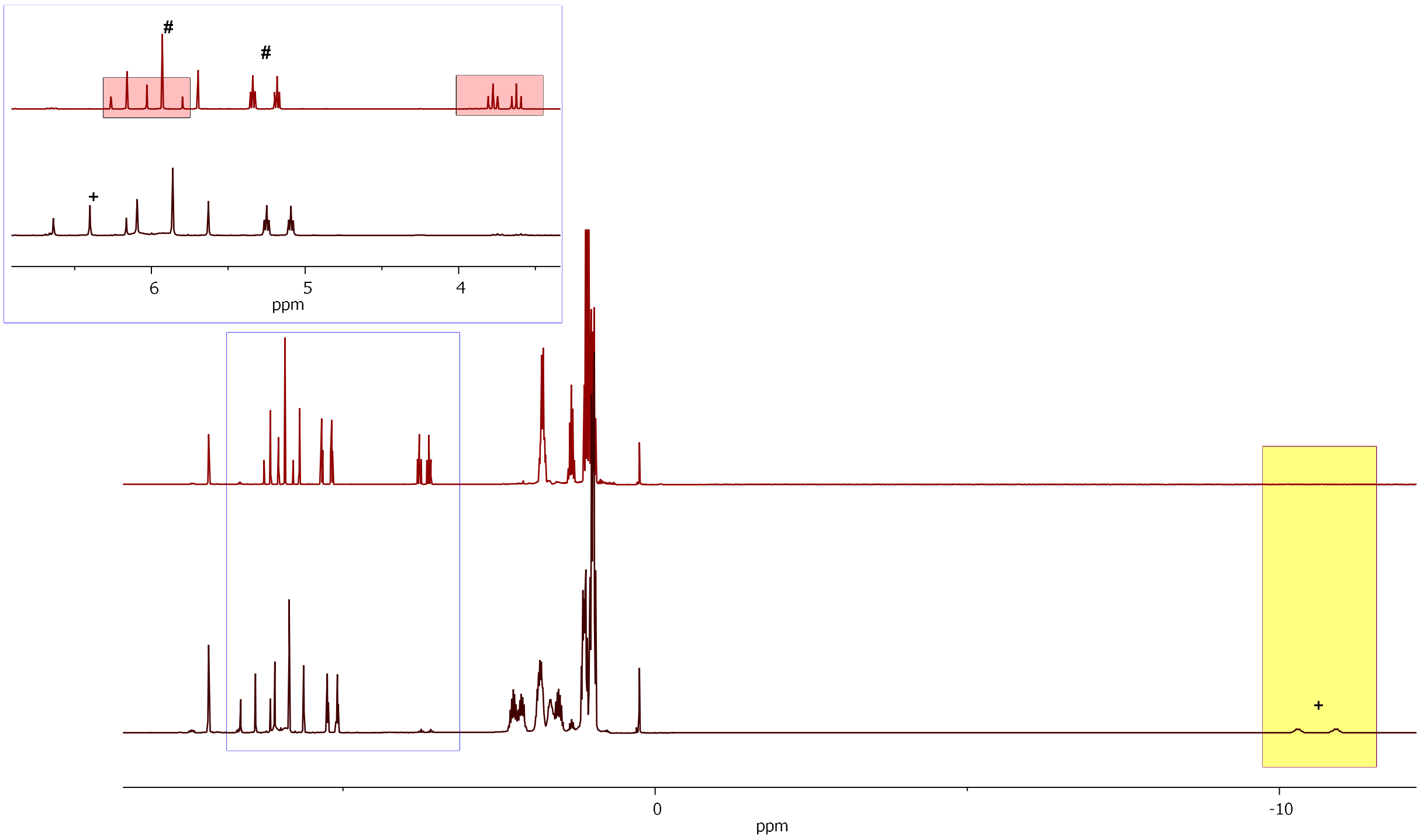

S14. ^{1}H NMR spectra of the reaction of $[Rh(H)(PEt_3)_3]$ (**1**) with an excess of enflurane (2.9 eq) (#) in C_6D_6 at room temperature after 20 minutes (below) and after 72 hours (above) showing the conversion of complex **8** (+) into and $CFH_2CF_2OCF_2H$ (highlighted in pink in the inset).

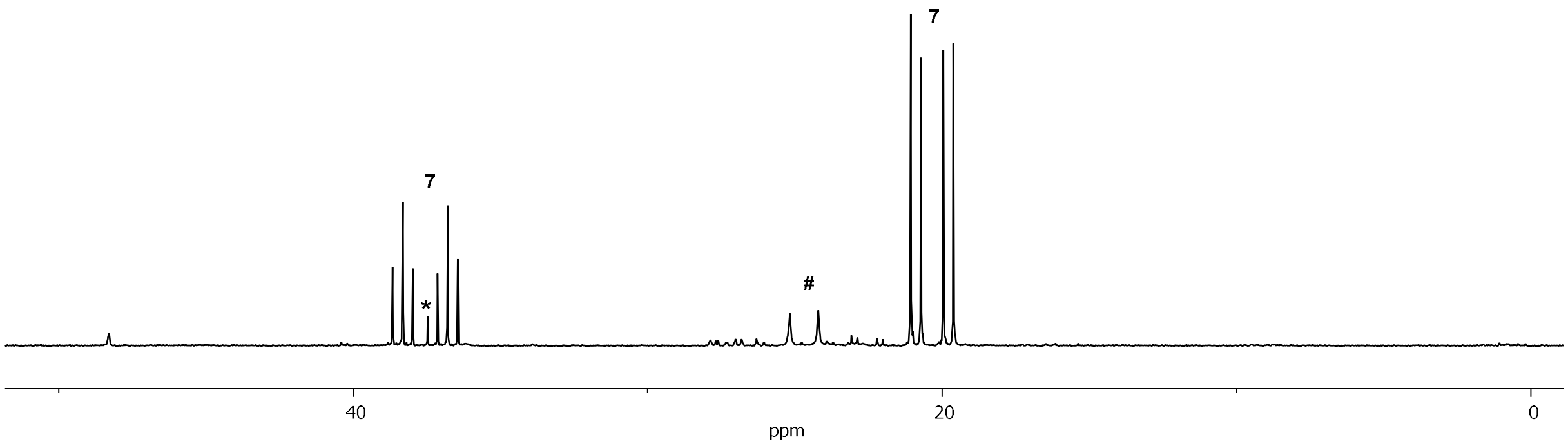

S15. $^{31}P\{^{1}H\}$ NMR spectrum of the reaction of $[Rh(H)(PEt_3)_3]$ (**1**) with an excess of isoflurane (+) in C_6D_6 after 1 hour at room temperature showing signals of complex **7**, **9** (*) and **10** (#).

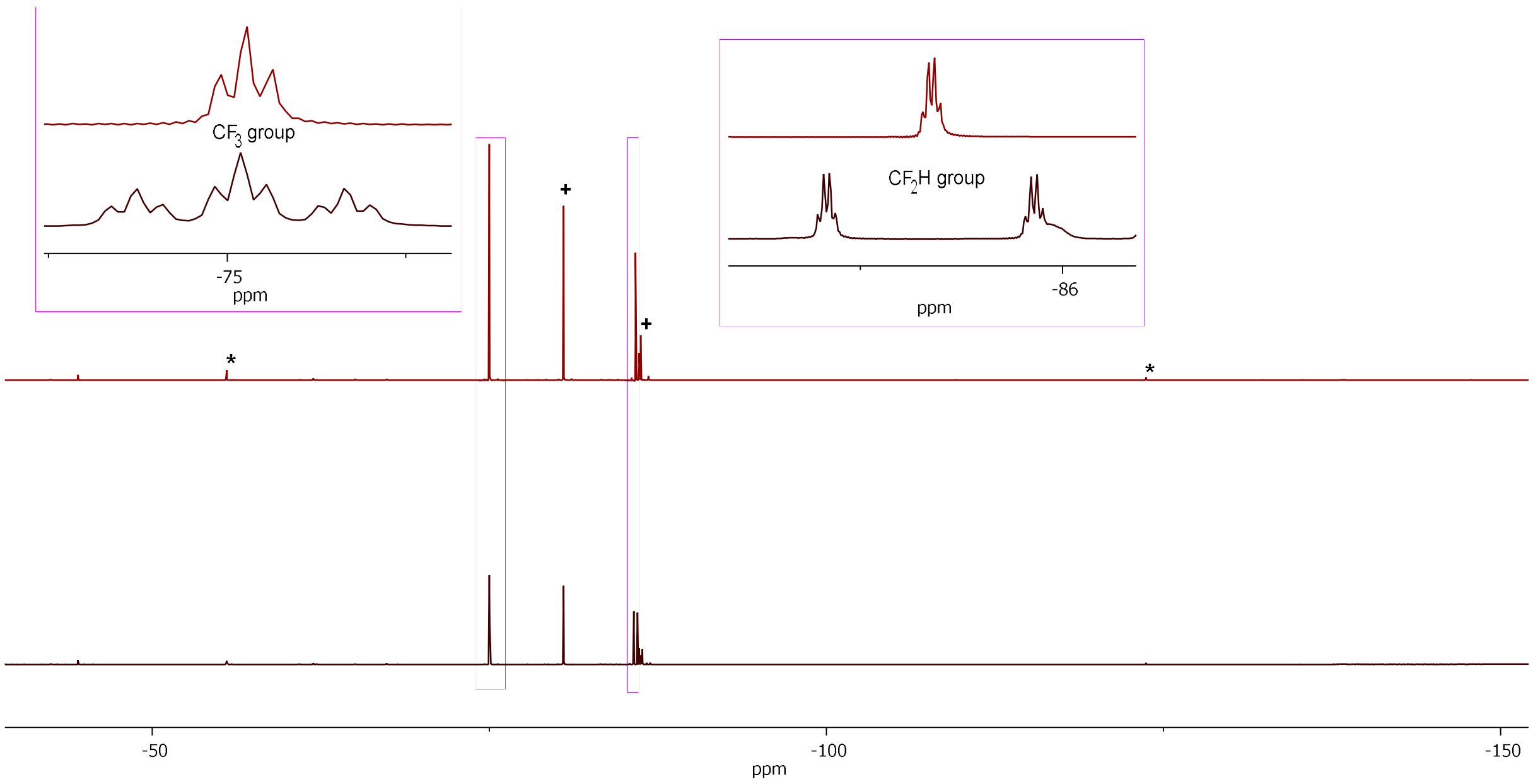

S16. ^{19}F NMR NMR spectrum (below) and $^{19}F\{^{1}H\}$ NMR spectrum (above) of the reaction of [Rh(H)(PEt_3)$_3$] (**1**) with an excess of isoflurane (+) in C_6D_6 after 1 hour at room temperature showing signals for $CF_3CH_2OCF_2H$, and **9** (*).

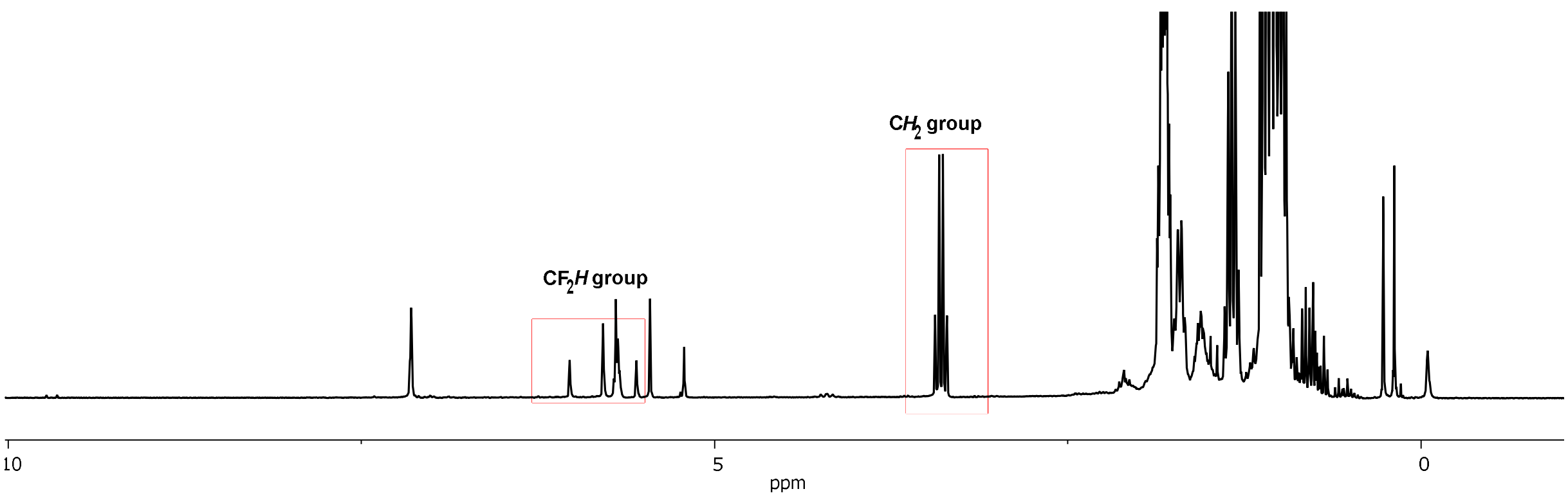

S17. ^{1}H NMR spectrum of the reaction of [Rh(H)(PEt_3)$_3$] (**1**) with an excess of isoflurane (+) in C_6D_6 after 1 hour at room temperature showing signals for $CF_3CH_2OCF_2H$.

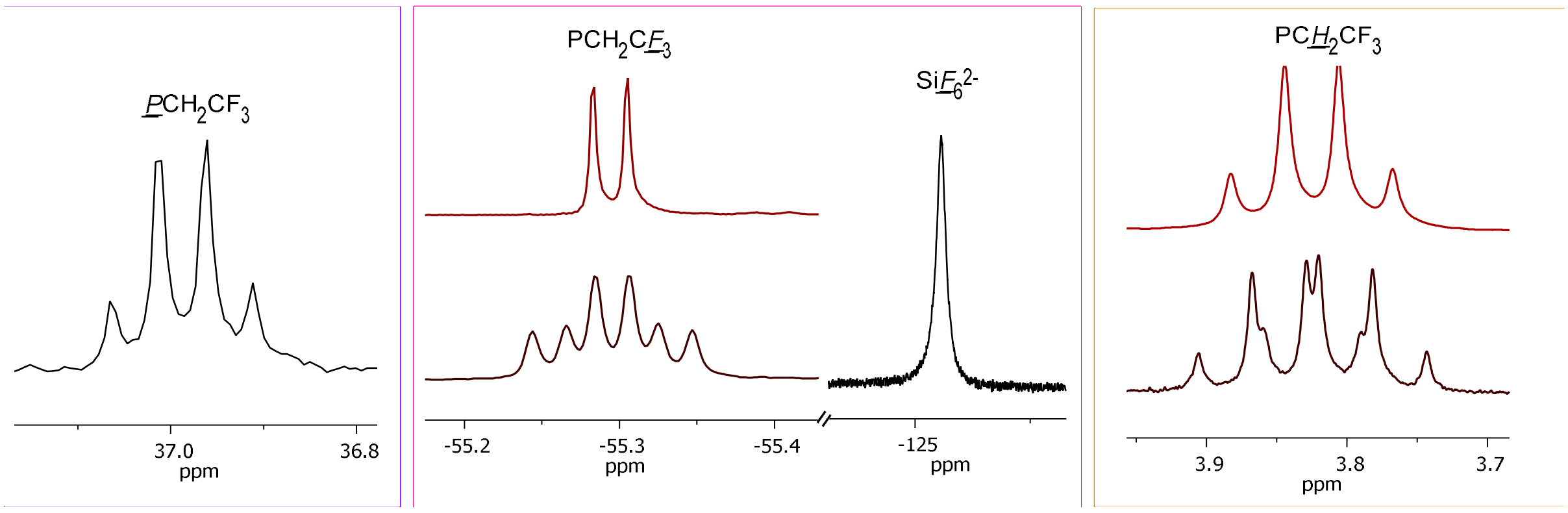

S18. Characteristic signals for the formation of compound **9**: $^{31}P\{^{1}H\}$ NMR spectrum (left); ^{19}F NMR spectrum (middle); and ^{1}H NMR spectrum (right). $^{19}F\{^{1}H\}$ NMR spectrum and $^{1}H\{^{31}P\}$ NMR spectrum are stacked above the relevant signals.

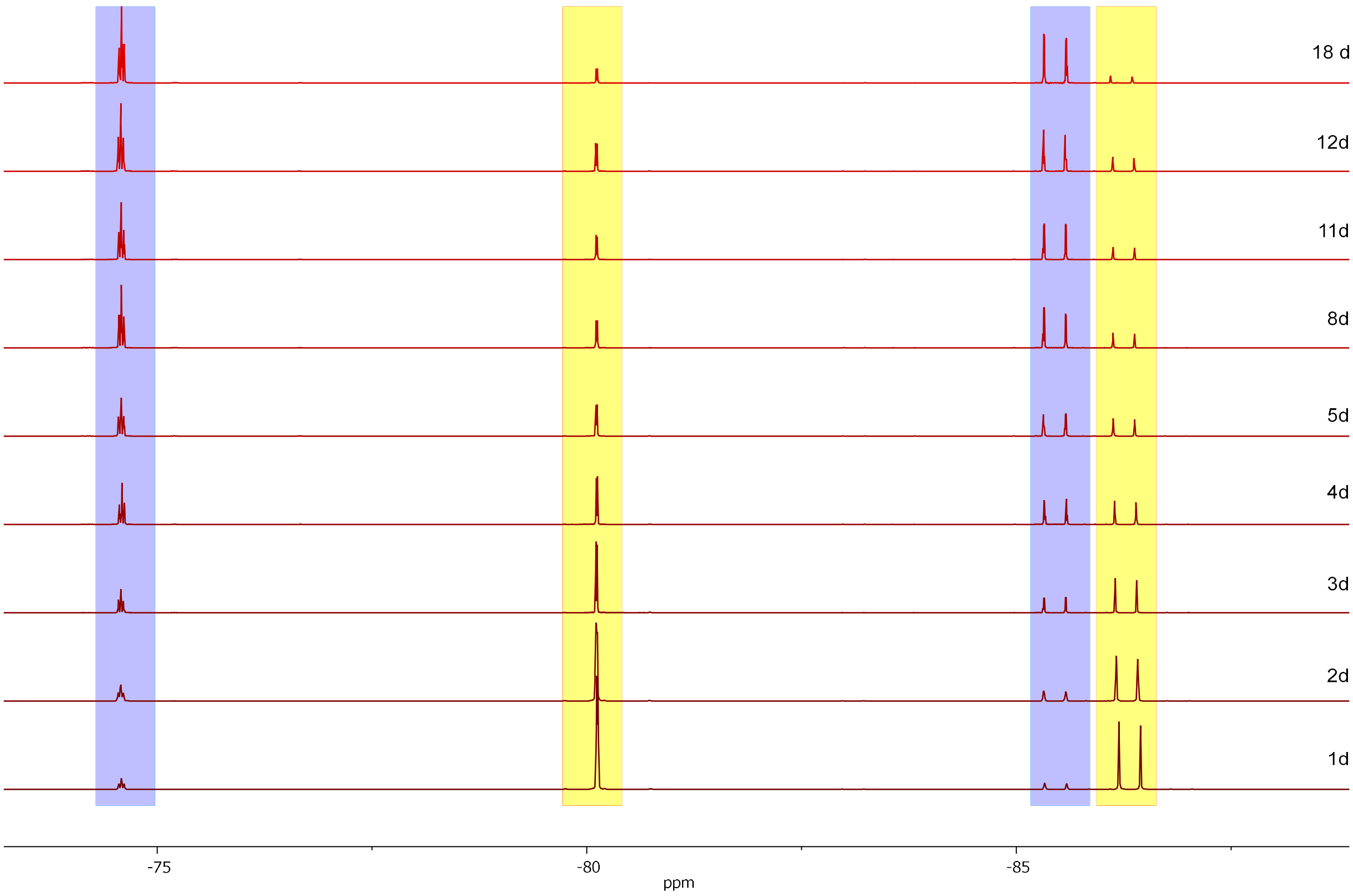

S19. ^{19}F NMR NMR spectrum of the catalytic reaction of isoflurane (yellow) with an excess of $HSiPh_3$ and $[Rh(H)(PEt_3)_3]$ (**1**) as catalyst in C_6D_6 over time at 58 °C showing the formation of $CF_3CH_2OCF_2H$ (purple).

3 Kinetic Study

For NMR monitoring experiments, the Rh(I) hydrido complex [Rh(H)(PEt_3)$_3$] (**1**) (0.087 mmol), enflurane (32 µL 0.26 mmol), trifluorotoluene (11 µL, 0.087 mmol) as internal standard and C_6D_6 (0.4 mL) were added to a JYoung NMR tube. ^{19}F NMR spectroscopy measurements were performed at different time points. The full conversion of **1** into the Rh(III) intermediate [Rh(H)(Cl)($CFHCF_2OCF_2H$)(PEt_3)$_3$] (**8**) was observed after 5 minutes. Formation of complex **7** and $CFH_2CF_2OCF_2H$ from **8** was then monitored. For these analyses, the parameter for recording the ^{19}F NMR spectra were adjusted and a relaxation time D1 = 30s was used (AQ = 2 s).

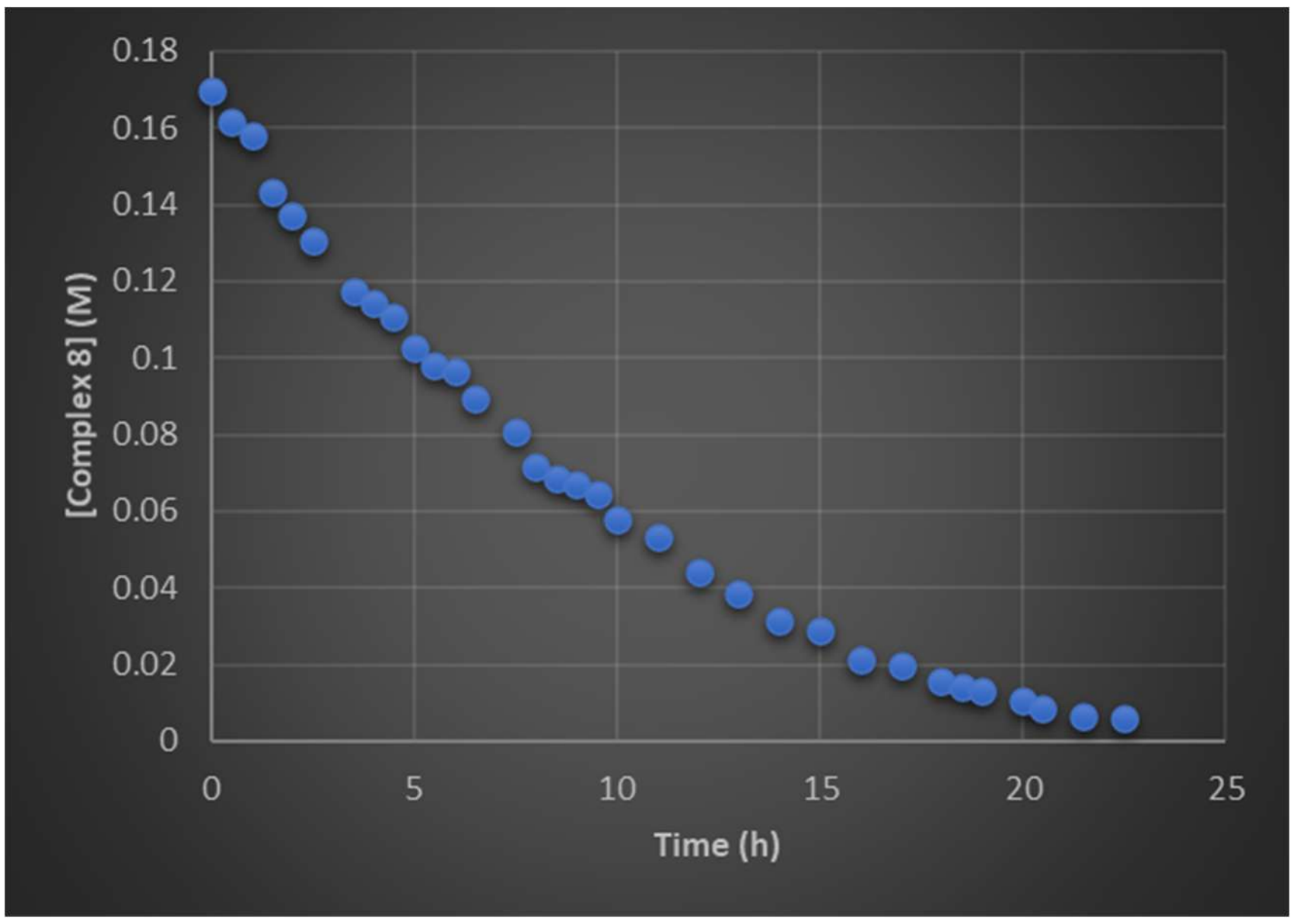

Figure S1. Plot of the concentration of complex **8** *vs.* time in the reaction of **1** with excess amount of enflurane. The concentration of complex **8** was determined based on the signal intensities in the ^{19}F NMR spectra. α,α,α-Trifluorotoluene was used as an internal standard.

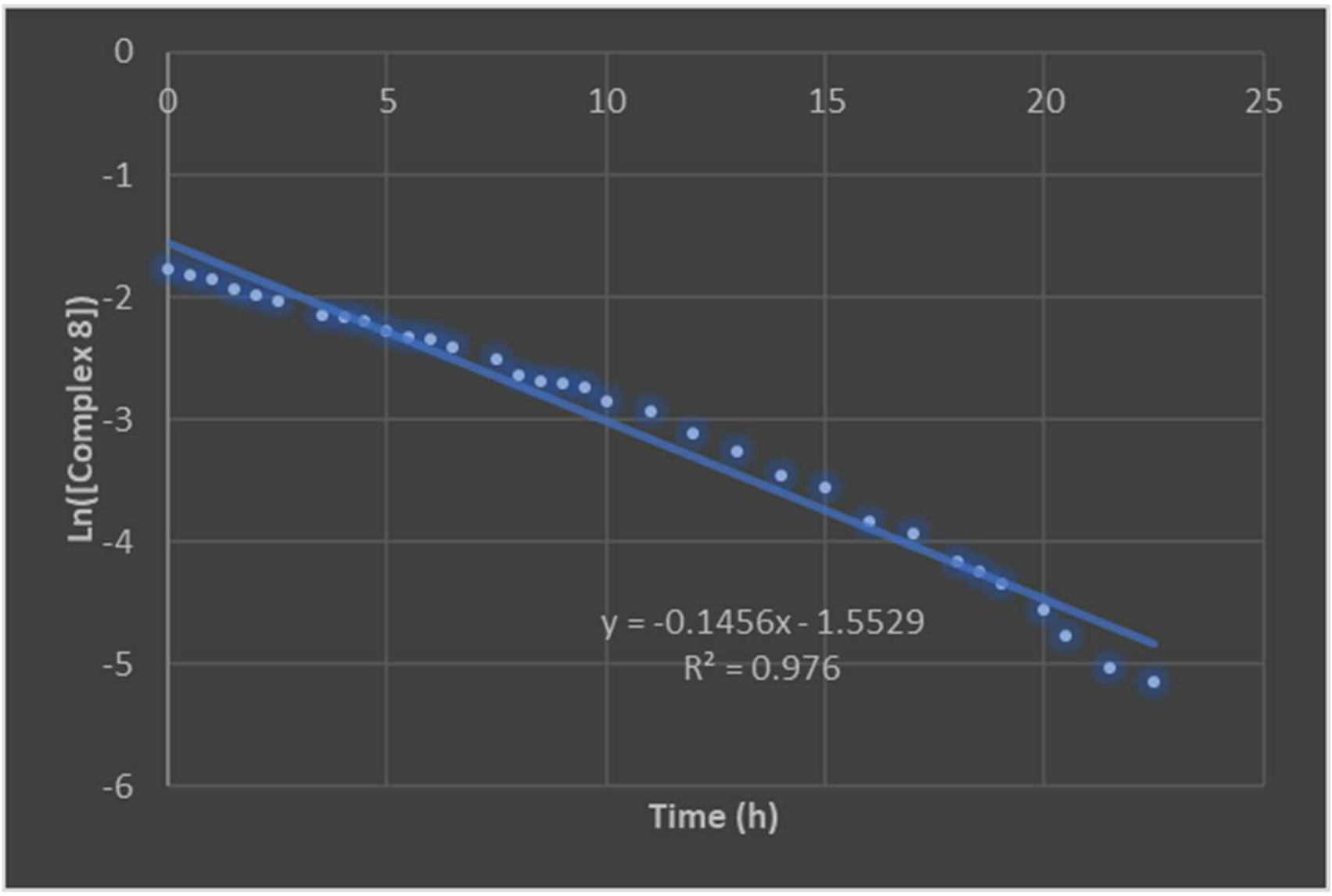

Figure S2. Plot of ln of the concentration of complex **8** *vs.* time in the reaction of **1** with excess amount of enflurane. The concentration of complex **8** was calculated based on the signal intensities in the ^{19}F NMR spectra. α,α,α-Trifluorotoluene was used as an internal standard. k= 0.1456 h^{-1} (k = 4.04 × 10^{-5} s^{-1}).

4 References

[1] D. D. Perrin, W. L. F. Armarego, *Purification of Laboratory Chemicals*, 3rd ed., Butterworth/Heinemann, London/Oxford, **1988**.

[2] T. Braun, D. Noveski, M. Ahijado, F. Wehmeier, *Dalton Trans.* **2007**, 3820-3825.

[3] R. D. Bagnall, W. Bell, K. Pearson, A. Jeater, *J. Fluorine Chem.* **1979**, *13*, 123-140.

[4] A. Foris, *Magn. Reson. Chem.* **2004**, *42*, 534-555.
